# Development of a Model Care Pathway for Myasthenia Gravis

**DOI:** 10.3390/ijerph182111591

**Published:** 2021-11-04

**Authors:** Anil babu Payedimarri, Matteo Ratti, Riccardo Rescinito, Alessandra Vasile, Deborah Seys, Hervé Dumas, Kris Vanhaecht, Massimiliano Panella

**Affiliations:** 1 Department of Translational Medicine (DIMET), Università del Piemonte Orientale, 28100 Novara, Italy; matteo.ratti@uniupo.it (M.R.); 10033325@studenti.uniupo.it (R.R.); 20034878@studenti.uniupo.it (A.V.); massimiliano.panella@med.uniupo.it (M.P.); 2 European Pathway Association, 3000 Leuven, Belgium; deborah.seys@kuleuven.be (D.S.); kris.vanhaecht@kuleuven.be (K.V.); 3 Department of Public Health and Primary Care, Leuven Institute for Healthcare Policy, KU Leuven, 3000 Leuven, Belgium; 4 UCB Pharma, 1630 Bulle, Switzerland; Herve.Dumas@ucb.com; 5 Department of Quality Management, University Hospitals Leuven, University of Leuven, 3000 Leuven, Belgium

**Keywords:** myasthenia gravis, critical pathway, care pathway, model pathway, integrated care pathway, clinical pathway, process flow, core activities, quality indicators, key interventions

## Abstract

Myasthenia Gravis (MG) is a chronic, life-lasting condition that requires high coordination among different professionals and disciplines. The diagnosis of MG is often delayed and sometimes misdiagnosed. The goal of the care pathway (CP) is to add value to healthcare reducing unnecessary variations. The quality of the care received by patients affected with MG could benefit from the use of CP. We conducted a study aimed to define an inclusive, comprehensive, and multidisciplinary CP for the diagnosis, treatment, and care of MG. The development of the model CP, key interventions, and process indicators is based on the literature review and 85 international MG experts were involved in their evaluation, expressing a judgment of relevance through the Delphi study. 60 activities are included in the model CP and evaluated by the MG experts were valid and feasible. The 60 activities were then translated into 14 key interventions and 24 process indicators. We believe that the developed model CP will help for MG patients to have a timely diagnosis and high-quality, accessible, and cost-effective treatments and care. We also believe that the development of model CPs for other rare diseases is feasible and could aid in the integration of evidence-based knowledge into clinical practice.

## 1. Introduction

Care Pathways (CPs) are a methodology used to help clinical teams in incorporating the best medical knowledge/evidences into clinical practice [1,2,3,4,5]. In brief, the goal of CPs is to add value to healthcare, reducing unnecessary variations through the standardization of the processes of care for a defined group of patients during a defined period [2,3,6]. Therefore, CPs could help healthcare teams to achieve better patient outcomes and quality of life, safer care, and more appropriate use of resources [7,8,9,10,11]. Usually, CPs are implemented for treating high volume and high-cost diseases/conditions, mainly in hospitals [12,13,14]. Nevertheless, the quality of the care received by patients affected with rare diseases could also benefit from the use of CPs. Many rare diseases are chronic, life-lasting conditions that require high coordination among different professionals and disciplines [15]. This could result in a lack of standardization of the care provided to the patients, poor clinical outcomes, reduced quality of life, and wasted resources [16].

Myasthenia Gravis (MG) is a rare autoimmune neuromuscular disorder, that affects 14–20 persons per 100,000 in the United States and 7–31 persons per 100,000 in Europe [17,18,19]. The diagnosis of MG is often slow and it is sometimes misdiagnosed [20]. There are significant variations in both medical treatment and settings of care, range from hospitals to long-term care facilities, and non-pharmacological treatments could be underused [21,22]. As a possible consequence, the quality of the life and the outcomes of the patients can be poor, and myasthenic crisis (MC) still affects 15–20% of MG patients, with a lethality of 3–8% [23,24].

Therefore, even though a significant number of clinical guidelines and algorithms for the diagnosis, treatment, and care of MG have been published [25,26,27,28], an optimal process of care has not yet been reached.

To this purpose, we conducted a study aimed to define an inclusive, comprehensive, and multidisciplinary clinical care pathway for the diagnosis, treatment, and care of MG.

## 2. Materials and Methods

**Figure 1:**
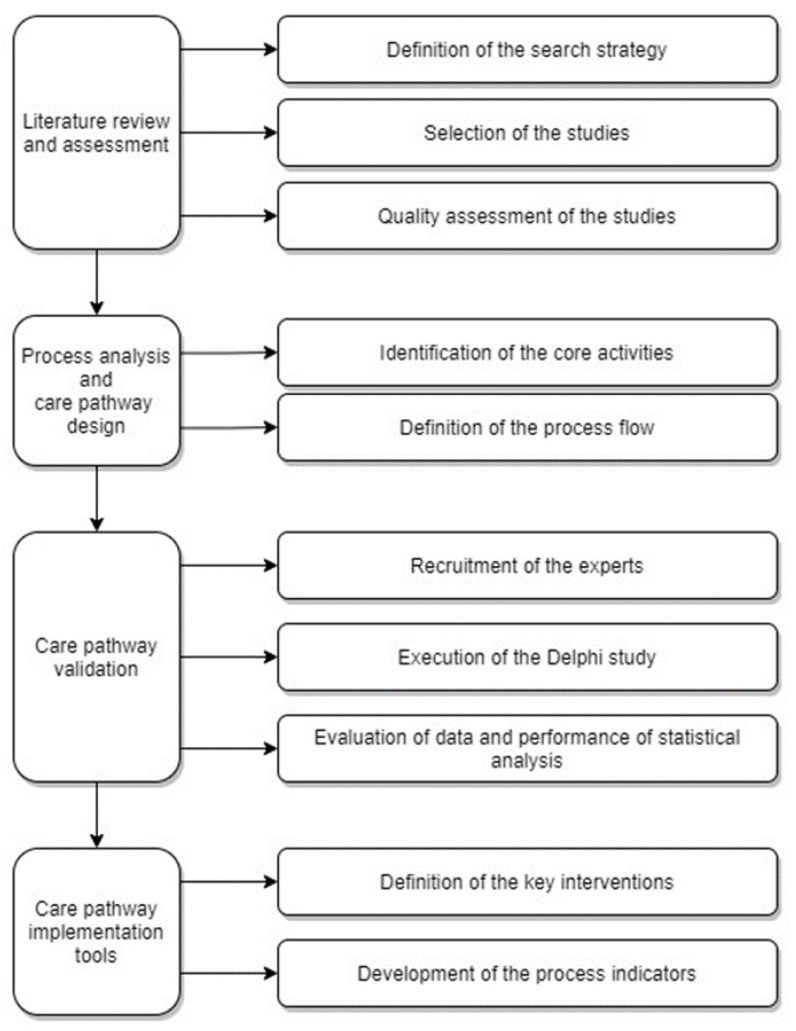
Flow diagram for the development of a model care pathway for MG.

The process for the development of the model care pathway is shown in Figure 1. In the first step a literature review was performed from the selected databases and the quality of studies were appraised. In the second step a care pathway was designed, in which the identified core activities were evaluated and clustered them in to process flow. In the third step, international MG experts were recruited, Delphi study were executed to evaluate and validate the core activities included in the care pathway, and the statistical analysis were performed to evaluate the responses of the MG experts. In the final step, care pathway implementation tools such as key interventions and process indicators were developed.

### 2.1. Literature Review and Assessment

#### 2.1.1. Definition of the Search Strategy

An extensive literature review was conducted by two researchers (A.V, AB. P). We searched for national and international guidelines, systematic reviews, reviews, clinical care pathways, process flows algorithms, clinical/critical/care indicators, outcome measures concerning the diagnosis, the assessment, the treatment, and the management of generalized and ocular myasthenia gravis, and of the myasthenic crisis in the following resources:

(i) Websites of international scientific societies: Myasthenia Gravis Foundation of America (MGFA) [https://myasthenia.org/], (accessed on 27 July 2020); Association of British Neurologists (ABN) [https://www.theabn.org (accessed on 27 July 2020)]; European Myasthenia Gravis Association (EuMGA) [http://www.eumga.eu (accessed on 27 July 2020)]; European academy of neurology (EAN)/ European Federation of Neurological Societies (EFNS) [https://www.ean.org (accessed on 27 July 2020)]; American Board of Electrodiagnostic Medicine (ABEM) [https://www.aanem.org]; American Association of Neuroscience Nurses (AANN) [https://aann.org]; American Academy of Neurology [https://www.aan.com (accessed on 27 July 2020)]; Japanese Society for Neuroimmunology (JSN) [http://www.neuroimmunology.jp (accessed on 27 July 2020)]; European Reference Network for Rare Neuromuscular Diseases (EURO-NMD) [https://ern-euro-nmd.eu (accessed on 27 July 2020)].
(ii) Public resources for evidence-based clinical practice guidelines: Guidelines international interwork (GIN) [www.g-i-n.net (accessed on 28 July 2020)]; National Institute for Health and Care Excellence (NICE) [https://www.nice.org.uk/ (accessed on 28 July 2020)]; Scottish Intercollegiate Guidelines Network [https://www.sign.ac.uk/ (accessed on 28 July 2020)]; Canadian clinical practice guidelines [https://joulecma.ca/cpg/ (accessed on 28 July 2020)]; Australian clinical practice guidelines [https://www.clinicalguidelines.gov.au/ (accessed on 28 July 2020)].
**(iii)**  Electronic databases: PubMed [https://pubmed.ncbi.nlm.nih.gov]; UpToDate [https://www.uptodate.com/]; Cochrane library [https://www.cochranelibrary.com/ (accessed on 30 July 2020)]; EMBASE [https://www-embase.com/ (accessed on 30 July 2020)]; EBSCO CINHAL [https://www.ebsco.com/products/research-databases/cinahl-database (accessed on 30 July 2020)].
(iv) Public resources for quality improvement indicators in healthcare: The Joint Commission [https://www.jointcommission.org/ (accessed on 31 July 2020)]; Agency for Health Care Research and Quality [https://www.qualitymeasures.ahrq.gov (accessed on 31 July 2020)]; Australian commission on safety and quality in health care [https://www.safetyandquality.gov.au/ (accessed on 31 July 2020)]; Canadian institute for health information [https://www.cihi.ca/ (accessed on 31 July 2020)]; Health quality Ontario [https://www.hqontario.ca/ (accessed on 31 July 2020)].

For PubMed, CINHAL, EMBASE, and Cochrane library we used the MESH terms: (a) ‘Myasthenia Gravis’ combined with ‘Guideline’ OR ‘Consensus Development Conference’ OR ‘Practice Guideline’ OR ‘Systematic reviews’ OR ‘Reviews’ AND ‘Patient care management’ AND ‘Outcomes’. (b) ‘Myasthenia Gravis’ combined with ‘Quality Indicators’. (c) ‘Myasthenia Gravis’ combined with ‘Critical Pathway’ OR ‘Clinical pathway’ OR ‘Care pathway’. We also adopted a snowballing search strategy that means that the reference lists from published original and review articles were searched manually to identify other possible eligible studies.

#### 2.1.2. Selection of the Studies

Two independent reviewers (A.V and AB. P) selected the literature according to the following inclusion criteria: (i) guidelines, systematic reviews, reviews, clinical care pathway process flow algorithms, and diagrams reporting clinical evidences for MG; (ii) articles describing measures of clinical performance (clinical/critical/care indicators) and outcomes for in and outpatients with MG; (iii) published until 1 November 2020; and (iv) published in English. As a first step, we evaluated the articles based on the title and the abstract. If the relevance of the article was unclear, or if the abstract was not available, we then appraised the full text. In case of disagreement, a third reviewer (D.S) was consulted. Finally, the reviewers (A.V and AB. P) thoroughly searched the included papers for all the possible clinical activities and outcomes related to in and outpatient’s assessment and management of MG. 

#### 2.1.3. Quality Assessment of the Studies

We evaluated the quality of the guidelines using the Appraisal of Guidelines for Research and Evaluation II (AGREE II), an internationally validated tool developed to assess the quality of practice guidelines with a focus on methodological development and transparency [29]. Two appraisers (M.R, R.R) were trained in the AGREE-II tool using the online tutorial [30]. According to AGREE-II, the researchers evaluated independently the six domains, the twenty-three key items, and the additional two global rating items for an “Overall Assessment” of the practice guideline. All the discrepancies were discussed between the appraisers and a specialist in the AGREE-II tool (K.V). The guidelines with an overall assessment score less than or equal to three were excluded.

### 2.2. Process Analysis and Care Pathway (CP) Design

#### 2.2.1. Identification of the Core Activities

We extracted any recommendations from the selected guidelines, and we evaluated the clinical care pathways presented in the selected review articles. All the identified activities were evaluated and classified according to the hierarchy of their level of evidence (LOE). The criteria of classification were adopted from EFNS [31]:

1. Class-A: evidence provided by a systematic review (SR) or randomized controlled trial (RCT).
2. Class-B: evidence provided by a review or observational study (prospective, retrospective, case-control) or controlled trial or RCT with limitations.
3. Class-C: evidence provided by an expert opinion (EO) or consensus or good practice point (GPP).

#### 2.2.2. Definition of the Process-Flow

First, to design a comprehensive CP for the diagnosis, the treatment, and the care of MG, we removed all the duplicated clinical activities. After adopting a unique format, we clustered the activities based on the following criteria: (i) clinical activities that were inevitably linked to each other, (ii) clinical activities that needed to be performed by a specific health care professional, and (iii) clinical activities that needed to be performed at a specific time point or within a specific time span of the care process.

Finally, we categorized the clusters into the following sub-processes: (i) diagnosis and assessment, (ii) pharmacological therapy, (iii) speech, swallowing, dental assessment and management, (iv) occupational, physical, respiratory assessment and management, (v) psychological assessment and management, (vi) lifestyle factors assessment and management and vii. MG crisis assessment and management. We used the io. draw tool [32] to draw the CP process flow diagram.

### 2.3. Care Pathway Validation

#### 2.3.1. Recruitment of the Experts

To evaluate and validate the newly defined model care pathway for MG, we recruited an international panel of doctors, experts in MG. The panel was composed of 85 clinical physicians (44 neuromuscular experts and 41 general neuro physicians) from the United States, Europe, and Japan. We enrolled the experts through a recruitment agency [33]. The recruitment criteria are reported in supplementary materials (S1).

#### 2.3.2. Execution of the Delphi Study

The study was conducted from November 2020 to February 2021. The Delphi method is a systematic and qualitative approach of forecasting that involves polling a group of experts via several rounds of questions. The Delphi method relies on subject matter experts to forecast the outcome of future scenarios, predict the likelihood of an event, or reach consensus on a particular topic [34]. The experts were asked to fill the questionnaire (online survey) that is reported in supplementary materials (S2). In brief, in the first round of the Delphi, the experts were asked: (i) to review the clinical activities of the model care pathway, checking for their validity and feasibility; (ii) to propose any additional clinical/ care activities that they believed to be essential for MG in- and out-patients, but not included in the proposed care pathway; (iii) to rank the activities for each sub-process, according to their importance (possible impact on patient’s clinical outcomes, quality of life, safety, and costs); (iv) to identify any potential bottlenecks when executing the CP; (v) to describe the current setting for execution of sub-process (e.g., hospitals, ambulatory care, etc.). In the second round of Delphi, we asked the experts to validate the updated model care pathway.

#### 2.3.3. Evaluation of Data and Performance of Statistical Analysis

To avoid overestimation due to non-respondents, we considered any incomplete part of the questionnaire as a negative answer [35]. To evaluate the results of the first round of Delphi, we defined a cut-off of 75% as a minimum level of consensus to consider a clinical activity as appropriate. We also added to the model pathway any new/missing clinical activities that were proposed by at least 25% of the experts. We considered approved the updated model care pathway if at least 95% of the experts did not propose any further modifications. The rank of clinical activities was calculated with a weighted score algorithm [36]. We classified the identified bottlenecks of the model care pathway according to the related activities. We considered relevant any bottlenecks that was reported by more than five experts.

We tested the proportions among the different regional groups of experts (EU, US, and JPN) with the Fisher-Freeman-Halton exact test. When a statistically significant difference was found, further post hoc Fisher tests were applied to detect the proportion which differed the most from the others. Bonferroni corrections of the significance level were applied based on the number of further tests needed. All statistical analysis were made with R ver. 4.1.0 and RStudio ver. 1.2.1335. 

### 2.4. Care Pathway Implementation Tools

#### 2.4.1. Definition of the Key Interventions

Key interventions are defined as a set of program elements that work together enhancing the health or affecting the outcomes in a specific group of patients. The adherence rates to key interventions are used to evaluate the real implementation of the care pathway into the current practice [37,38].

To identify the key interventions, we combined the LOE of each clinical/care activity (as it was measured before) with the importance of the same activities attributed by the experts (in the Delphi study). This final grade let us to re-rank the clinical activities of each sub-process, where the best grade was attributed to the clinical activities that combined the highest LOE with the highest clinical importance.

We listed the activities according to their final grade. We selected as a key intervention each activity that was in the first tertile for every sub-process. During this process, we also grouped similar/related activities in a new multiple sub-component’s key intervention. Each key intervention was described according to the model developed by the European Pathway Association (E-P-A) [38,39].

#### 2.4.2. Development of the Process Indicators

We defined at least one process indicator measuring the performance of each key intervention. This was made according to the methodology for developing a core performance measure of processes by the joint commission [40] and the Agency for Health Care Research and Quality [41]. 

The final list of the key interventions and of the process indicators was validated by the experts in the second round of the Delphi study.

## 3. Results

### 3.1. Literature Review and Assessment

**Figure 2:**
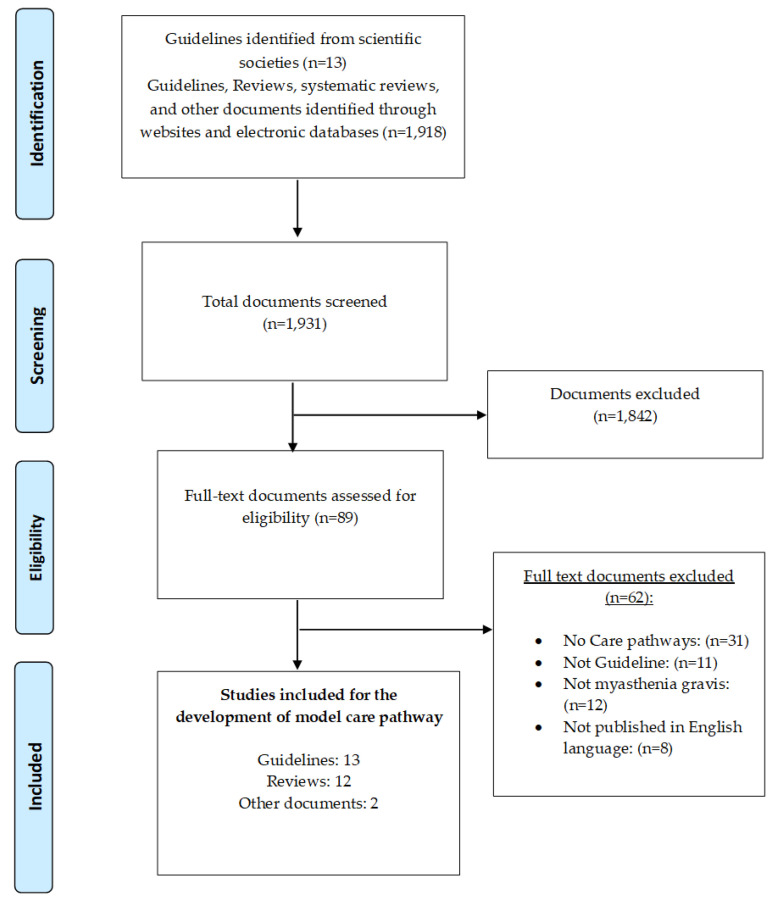
Flow Diagram of article selection process.

From the searched databases we identified 1931 records. After the manual screening, we excluded 1842 articles that were not relevant. Therefore, 89 full-text records were assessed for eligibility: 62 did not meet the inclusion criteria and were excluded. The remaining 27 papers included (Figure 2) 13 guidelines (reporting clinical evidences) [25,26,42,43,44,45,46,47,48,49,50,51,52], 11 reviews (presenting process flows, pathways, and algorithms of integrated care) [27,28,53,54,55,56,57,58,59,60,61], 1 clinical metrics article (with tools to measure outcomes and process indicators) [62], 1 manual reporting MGFA multi-disciplinary practice recommendations [63] and 1 document with benchmarking information (EURO-NMD network) [64].

The characteristics and the quality assessment of the included guidelines are described in the supplementary materials (Table S3). In brief, 7 guidelines were developed by US scientific societies (AAN, AANN, AAEM, and AANEM); 3 were developed in Europe by EFNS; 2 were developed in the UK by ABN and by the UK-multispeciality working group, and the last guideline was developed in Japan by JSN.

The characteristics of reviews that are presenting proposed and/or suggested process flows, pathways, and algorithms of integrated care for MG and other included documents are shown in the supplementary materials (Table S4).

### 3.2. Process Analysis and CP Design

From the literature, we identified and retrieved 243 clinical activities for the diagnosis, the treatment, and the care of MG patients: 183 duplicates were then removed, and the final list consisted of 60 clinical activities. Of those, 8 activities were graded as Class-A evidences, 18 as Class-B evidences, and 34 as Class-C evidences. 

The 60 activities were then modelled in a process flow according to their clinical dimension (e.g., diagnostic, therapeutic, etc.) and to their level of hierarchy in the process of care. The resulting overall comprehensive MG model CP is shown in the supplementary materials (Figure S5). In brief, 10 clinical activities were included in the diagnostic process; 12 in the pharmacological management process; 6 in the management process of speech, swallowing, and dental needs; 13 in the occupational, physical, and respiratory management processes; 8 in the care process of psychological needs; 6 activities constituted the lifestyle management process; and the last 5 in the process of assessment and management of the myasthenic crisis.

### 3.3. CP Validation

The newly defined model pathway was evaluated by the international experts [65]. 

The panel was composed of 85 physicians: 59 experts (69.4%) had more than 10 years of experience in treating MG patients; 20 (23.5%) had between 5 and 10 years of experience, and 6 (7.1%) had less than five years of experience. About the patients’ volumes: 41 doctors (48.2%) treated between 5 and 20 patients monthly, 37 (43.5%) treated more than 20 patients and 7 (8.2%) treated less than five patients on a regular monthly basis.

Most of the experts were working in a neuromuscular specialty center (*n* = 58, 68.2%), mainly in academic institutions (*n* = 50, 58.8%). Some experts described their working places more in detail, including as a private center (*n* = 25, 29.4%), a private not-for-profit (*n* = 3, 3.5%), a public institution (*n* = 10, 11.8%) or as another different typology of organization (*n* = 9, 10.6%). 

The patients were treated mainly in out-patient clinics (78.9%) and in hospitals (65.9% neurology wards and 21.2% day-care), followed by physiotherapy and rehabilitation centers (10.6%), and facilities for speech therapy and for occupational therapy (7.1% and 5.9% respectively).

As shown in Table 1, we observed a high level of agreement among the experts both for completeness (from 85.9% to 95.3%) and for appropriateness (from 83.5% to 89.4%) of the proposed model care pathway. We did not observe any differences related to the provenience of the experts, except for the speech, swallowing, and dental management process that was considered incomplete by the Japanese experts (*p* = 0.02896; post-hoc *p* = 0.01642).

We compared the experts’ opinions with the LOE previously attributed from the literature and found no significant differences among the grades. In fact, approximately 3% of the experts considered the activities to be inappropriate regardless of the grade (2.7% of experts for class A and C activities, 3.2% for class B). 

In Table 2 we described and analysed the settings where the patients were treated along the process of care. Overall, patients’ diagnosis and assessment and the administration of the pharmacological therapies were performed equally in ambulatory care (51.8% and 55.3%) and in hospital wards (45.9% and 42.1%) with significant regional differences. In fact, 22.9% of European experts diagnosed and administered treatment to their patients in ambulatory settings (*p* < 0.0001 and *p* < 0.0001 respectively). On the contrary, European patients mostly received their diagnosis in hospitals (*p* = 0.001543) whereas in the United States only 23.3% of the experts used the hospitals for the pharmacological treatment (*p* = 0.002065). Occupational therapy activities were carried out in nursing homes more frequently in the United States (25.6%) and Japan (28.6%) than the EU (5.7%) (*p* = 0.04046). Lastly, patient lifestyle support was mainly given in ambulatory care in the United States (83.7%) and in Japan (85.7%) when compared to EU (57.1%) (*p* = 0.0235). In overall, 25 potential bottlenecks emerged out of the 60 activities (41.6%). Of those, 8 were considered relevant because were reported by more than 5 experts.

### 3.4. CP Implementation Tools

**Figure 3:**
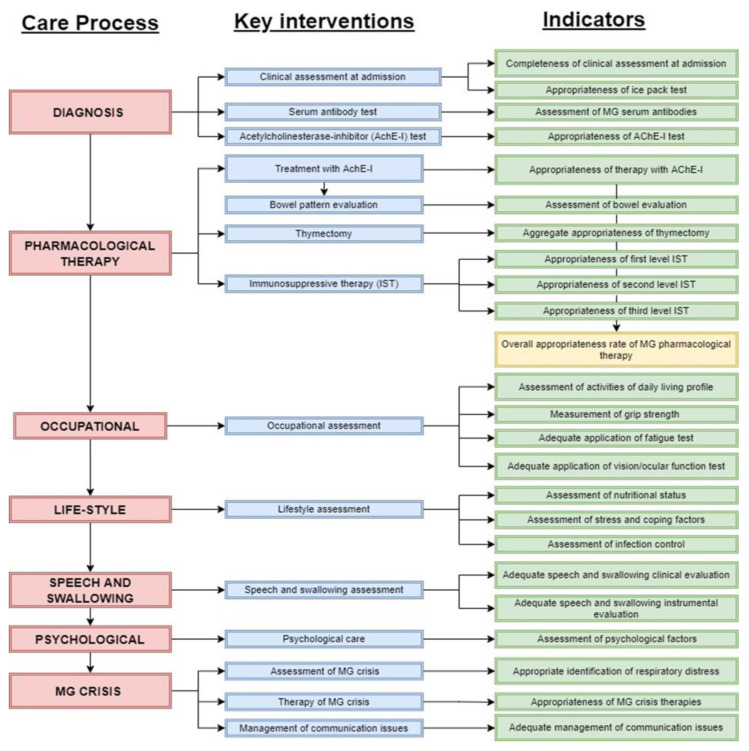
Care pathway implementation tools.

Figure 3 shows the list of the key interventions and of the relative process indicators. We identified 14 key interventions as follows: diagnostic process (*n* = 3); pharmacological therapy (*n* = 4); speech and swallowing (*n* = 1); occupational, physical, and respiratory (*n* = 1); psychological assessment (*n* = 1); lifestyle assessment (*n* = 1); and myasthenic crisis (*n* = 3) assessment and management processes. An example of a key intervention is fully described in the supplementary materials (Figure S6). The 24 process indicators included the measurement of the performance of the diagnosis and assessment (4 indicators), the pharmacological therapy (7 indicators), the speech and swallowing assessment (2 indicators), the occupational assessment and management (4 indicators), the psychological assessment (1 indicator), the life-style assessment (3 indicators) and the assessment and management of myasthenic crisis (3 indicators) processes. An example of an indicator is fully described in the supplementary materials (Figure S7).

## 4. Discussion

The development of CPs is a process that starts incorporating the best medical literature into clinical practice [66]. Often, several guidelines are produced for the most common diseases, frequently presenting conflicting evidence [67]. Therefore, selecting the most appropriate evidence to be included in CPs can be complex [68]. This is also a continuous process because medical literature is continuously updated, and CPs need to be updated accordingly [69].

When developing the CP for MG, our findings showed a different scenario. In fact, the number of clinical practice guidelines published for MG during the last ten years was significantly lower than the similar disease condition [70,71] and most of them are presented same similar clinical contents.

Usually, core activities are based on the strongest evidence [72]. This was not the case for MG, because only a few of the core activities were derived from class-A evidence, whereas most of them were based on expert opinion. This could be a critical problem when implementing operational care pathways^(b)^ locally, because local teams should consider that they are incorporating expert opinion rather than objective data into their clinical practice, and after the implementation professionals tend not to consider any more the original quality of the evidences [73].

In our study, there was a high level of agreement between experts both for the completeness of the newly developed model pathway (process flow) and for the appropriateness of the included clinical/care activities, regardless of their LOE (class-A, B, and C evidence). Even if we cannot conclude that the experts gave the same value to practices based on opinions more than on evidence, again we think that this issue could affect the quality of local CPs. Therefore, we believe that the relationship between the objective level of evidence and how they are perceived by healthcare professionals should be further studied in other settings.

The largest variation in experts’ opinions was around the settings for carrying out the diagnostic activities and for the patients’ treatment. In the United States, these processes were mainly based in ambulatory settings, whereas in Europe they were executed generally in hospitals. We can reasonably assume that the observed differences reflect the different models of the healthcare systems (private vs. public) [74,75], as it has been also shown in the studies measuring variations in the costs for the treatment of MG patients [76]. Since the local implementation of CPs combines the most appropriate care with the most appropriate use of resources, this could be a further critical issue for applying the MG model CP in real life [77,78].

Teamwork is a key component for the successful implementation of CPs [79,80,81]. To better organise their work, CP teams need information on performance of their core processes before and after implementation of the CPs [35]. Therefore, an effective model care pathway^(a)^ should also include the most appropriate metrics, process performance, and/or quality indicators derived from the literature [82]. In general, such core performance measures already exist in the medical literature [40,41]. This was not the case when looking for the core performance measures for MG. Therefore, we autonomously developed new quality indicators and metrics. This development process was driven by the information gathered from the experts, adopting a model that combined the importance of each process with the related risk of bottlenecks and/or its expected impact on patients’ outcomes. Even if we adopted a rigorous process design, nevertheless our indicators have not been validated yet. This is a further major limitation for the future implementation of the operational CPs for MG. Therefore, we draft a study to test the validity and the generalizability of the set of core performance measures and quality indicators.

- Model CP: The model care pathway is based on the available international and national evidence. It is not specific to any organization.
- Operational/Local CP: The operational pathway is the pathway developed by a specific organization based on the information from the model care pathway and specific organization characteristics (available competences, resources, etc.) Because of the differences between organizations, this pathway is organization specific.

## 5. Conclusions

In conclusion, based on our findings, we believe that the newly developed model CP for MG could help clinical teams to better organize a timely diagnosis and high-quality, accessible, and cost-effective treatments and care for their patients. Our results also suggest that future research on MG should pursue high-quality evidence (more primary studies with robust design are needed), a shared validated set of core performance measures (among different centers and countries), and the integration of the preference of patients in the CP. There is also the need to develop more guidelines covering non-pharmacologic therapies; the appropriate settings for treating MG patients should also be studied more in-depth.

The goal of our study was to define a model CP for MG. The development of CPs for rare diseases is strongly recommended in the literature, because for these conditions the knowledge is often scattered, and patients access to care and treatment could be heterogeneous and difficult [83,84]. Our experience demonstrated that the development of model CP for a rare disease is feasible and could be of help in integrating evidence-based knowledge into clinical practice.

## Figures and Tables

**Table 1:** Care pathway process evaluation: descriptive and statistical analysis.

Sub Process	Clinical Activities (*n* = 60)	LOE			Expert Opinions							
					Completeness Rates				Appropriateness Rates			
		A	B	C	Overall (*n* = 85)	USA (*n* = 43)	EU (*n* = 35)	JPN (*n* = 7)	Overall (*n* = 85)	USA (*n* = 43)	EU (*n* = 35)	JPN (*n* = 7)
(1) Diagnostic process	10	-	3	7	95.3%	95.3%	97.1%	85.7%	84.7%	88.4%	80.0%	85.7%
(2) Pharmacological management process	12	6	3	3	90.6%	88.4%	97.1%	71.4%	83.5%	83.7%	82.9%	85.7%
(3) Speech, Swallowing and Dental management process	6	-	2	4	90.6%	93.0%	94.3%	57.1% *	88.2%	88.4%	91.4%	71.4%
(4) Occupational, Physical and Respiratory management process	13	-	2	11	92.9%	95.3%	91.4%	85.7%	89.4%	88.4%	91.4%	85.7%
(5) Psychological management process	8	-	-	8	85.9%	81.4%	88.6%	100.0%	88.2%	86.0%	91.4%	85.7%
(6) Life-style management process	6	-	2	4	90.6%	88.4%	94.3%	85.7%	89.4%	90.7%	88.6%	85.7%
(7) MG crisis management process	5	2	2	1	89.4%	90.7%	91.4%	71.4%	89.4%	93.0%	85.7%	85.7%
Average of sub processes					90.8%	90.4%	93.5%	79.6%	87.6%	88.4%	87.3%	83.7%

Proportions among the three groups were analysed with Fisher-Freeman-Halton exact test. * *p* = 0.02896, post hoc test *p* = 0.01642 (Bonferroni inflated alpha = 0.01667).

**Table 2:** Care pathway setting evaluation: descriptive and statistical analysis.

Sub Process	Setting																			
	Ambulatory Care				Hospital Day Care				Hospital in-Patient				LTC (Nursing Home)				LTC (Rehabilitation)			
	*Overall (n = 85)*	*USA* (*n* = 43)	*EU* (*n* = 35)	*JPN* (*n* = 7)	*Overall (n = 85)*	*USA* (*n* = 43)	*EU* (*n* = 35)	*JPN* (*n* = 7)	*Overall* (*n* = 85)	*USA* (*n* = 43)	*EU* (*n* = 35)	*JPN* (*n* = 7)	*Overall* (*n* = 85)	*USA* (*n* = 43)	*EU* (*n* = 35)	*JPN* (*n* = 7)	*Overall* (*n* = 85)	*USA* (*n* = 43)	*EU* (*n* = 35)	*JPN* (*n* = 7)
(1) Dx	*51.8%*	72.1%	22.9% ^1^	71.4%	*15.8%*	16.3%	17.1%	0.0%	*45.9%*	30.2%	68.6% ^2^	28.6%	*2.4%*	4.7%	0.0%	0.0%	*4.7%*	7.0%	2.9%	0.0%
(2) Ph Tx	*55.3%*	81.4%	22.9% ^3^	57.1%	*20.0%*	14.0%	31.4%	0.0%	*41.2%*	23.3% ^4^	60.0%	57.1%	*5.9%*	9.3%	2.9%	0.0%	*3.5%*	4.7%	2.9%	0.0%
(3) SSD	*56.5%*	65.1%	45.7%	57.1%	*22.4%*	18.6%	25.7%	28.6%	*41.2%*	30.2%	51.4%	57.1%	*20.0%*	27.9%	8.6%	28.6%	*18.8%*	18.6%	14.3%	42.9%
(4) OPR	*63.5%*	74.4%	54.3%	42.9%	*22.4%*	16.3%	25.7%	42.9%	*23.5%*	18.6%	28.6%	28.6%	*17.6%*	25.6%	5.7% ^5^	28.6%	*20.0%*	23.3%	11.4%	42.9%
(5) PAM	*72.9%*	79.1%	62.9%	85.7%	*18.8%*	14.0%	25.7%	14.3%	*25.9%*	20.9%	34.3%	14.3%	*17.6%*	23.3%	8.6%	28.6%	*14.1%*	14.0%	14.3%	14.3%
(6) LAM	*72.9%*	83.7%	57.1% ^6^	85.7%	*21.2%*	14.0%	28.6%	28.6%	*24.7%*	25.6%	25.7%	14.3%	*22.4%*	30.2%	11.4%	28.6%	*17.6%*	16.3%	20.0%	14.3%
(7) M crisis	*24.7%*	25.6%	20.0%	42.9%	*15.3%*	14.0%	20.0%	0.0%	*72.9%*	72.1%	74.3%	71.4%	*5.9%*	7.0%	2.9%	14.3%	*4.7%*	2.3%	5.7%	14.3%
Average	*56.8%*	68.8%	40.8%	63.3%	*19.3%*	15.3%	24.9%	16.3%	*39.3%*	31.6%	49.0%	38.8%	*13.1%*	18.3%	5.7%	18.4%	*11.9%*	12.3%	10.2%	18.4%

Proportions among the three groups were analysed with Fisher-Freeman-Halton exact test. (1) *p* ≤ 0.0001; (2) *p* = 0.001543; (3) *p* ≤ 0.0001; (4) *p* = 0.002065; (5) *p* = 0.04046; (6) *p* = 0.0235. The post hoc analysis confirmed the differences except for n°5 (*p* = 0.02005 where Bonferroni inflated alpha was set at 0.01667). Dx: Diagnosis; Ph Tx: Pharmacological therapy; SSD: Speech, Swallowing and Dental; OPR: Occupational, Physical and Respiratory; PAM: Psychological.

## Supplementary Materials

The following are available online at https://www.mdpi.com/article/10.3390/ijerph182111591/s1, S1: Recruitment criteria for MG experts, S2: MG Questionnaire Delphi study, Table S3: Characteristics of included guidelines across the USA, Europe, Japan, and their overall quality scores by AGREE-II tool, Table S4: Characteristics of included reviews and other documents, Figure S5: Model pathway of MG, Figure S6: Example of a detailed description of a Key intervention, Figure S7: Example of a detailed description of a process indicator.
