# Supplementary material for: Development of a Model Care Pathway for Myasthenia Gravis
Source: Int J Environ Res Public Health. 2021 Nov 4;18(21):11591. doi: 10.3390/ijerph182111591 (PMC8582978; doi:10.3390/ijerph182111591)
Supplement: Supplementary file 1 [file ijerph-18-11591-s001.zip › S1_ Experts_Recruitment criteria_MG.pdf]

S1: Recruitment criteria for MG experts

A.) The recruitment criteria of the Neuromuscular experts were the following:

1. Qualified and currently practising as a Neurology Specialist in Neuro-Muscular Disorders (with key interest in MG)
2. MG Neuro Specialist should be at least one of the following:
    a. Chair/board member/expert advisor to a scientific or advocacy committee for an MG advocacy organization
    b. Or member of a MG medical association, MG professional society or MG scientific network
    c. Or a chair/speaker at a non-promotional, scientific MG conference/ meeting/ symposium (national/international)
    d. Or author/co-author of MG publication in a peer-reviewed journal within the last 3 years
    e. Or MG speaker (including lectures or poster presentations) at Neuro international/national/regional conferences.
    f. Or actively involved in a MG clinical research program
3. Must spend at least 30% of time in a hospital with a specialized neurology (MG) department or in Neuro-Muscular (MG) excellence/reference centre.
4. Spends at least 50% of time directly with patients.
5. Practising for between 3 years and not being fully retired
6. Responsible for managing at least:
    a. > 9 adults (generalized MG patients) on a yearly basis in US/JPN.
    b. > 5 adults (generalized MG patients) on a yearly basis in EU.

B.) The recruitment criteria of the General (or peripheral) Neurologist / Neurophysician experts were the following:

1. Mix of General Neurologist from community vs. academic based institutions, private practice vs. salaried employee of larger healthcare system.
2. Spends at least 50% of time directly with patients.
3. Practising for between 3 years and not being fully retired
4. Responsible for managing at least: > 5 adults, generalized MG patients on a yearly basis in US/JPN or > 2 adults, generalized MG patients on a yearly basis in EU.
