# Supplementary material for: Development of a Model Care Pathway for Myasthenia Gravis
Source: Int J Environ Res Public Health. 2021 Nov 4;18(21):11591. doi: 10.3390/ijerph182111591 (PMC8582978; doi:10.3390/ijerph182111591)
Supplement: Supplementary file 1 [file ijerph-18-11591-s001.zip › S2_MG Questionnaire_Delphi study.pdf]

* Required Information

Model Pathway for benchmarking the treatment of Myasthenia Gravis in the adult

Introduction

The aim of this project is to build a comprehensive and integrated Model Pathway for the treatment of Myasthenia Gravis (MG) disease in the adult. This will be achieved through the study of existing variations in current practice for the treatment of MG worldwide that will include different phases:

1- The definition of evidence-based best practices and indicators gathered from the literature search and expert opinions.
2- The identification of the potential gap between current practice and evidence.
3- Direction to bridge identified gaps.

The goal of this questionnaire is to gather your opinion in order to validate the process of care for myasthenia gravis that was designed by our research team based on existing guidelines and reviews.

2. Q1 Please select your area of profession/expertise
(Important Note: This question is compulsory unless your preferred answer option is greyed out. In that case, you can leave the question unanswered and click on the Next button to submit) (Select one option)

- ○ General (or peripheral) Neurologist / Neuro Physician
- ○ Neuro-muscular expert
- ○ Other (Please specify) ___________

NOTE : IF ANSWER TO **Q2** is
General (or peripheral) Neurologist / Neuro Physician Go to Page No. 3
Neuro-muscular expert Go to Page No. 3
Other (Please specify) Go to Page No. 2
If Did Not Answer Then Stop, you have finished the survey

NOTE : Answer the below question only if answer to Q#2 is General (or peripheral) Neurologist / Neuro Physician OR Neuro-muscular expert

*** 3. Are you working in Neuro-muscular disease expert centre? (Select one option)**

- ○ Yes
- ○ No

NOTE : Answer the below question only if answer to Q#2 is General (or peripheral) Neurologist / Neuro Physician OR Neuro-muscular expert

*** 4. Please select which type of institution are you working?**

Note: you can select more than one option.

- [ ] Academic
- [ ] Non-academic
- [ ] Private
- [ ] Private not-for-profit
- [ ] Public
- [ ] Other (Please specify) _______________

NOTE : Answer the below question only if answer to Q#2 is General (or peripheral) Neurologist / Neuro Physician OR Neuro-muscular expert

*** 5. Please select the setting where you are working**

Note: you can select more than one option.

- [ ] Out-patient clinic
- [ ] Day Hospital
- [ ] Hospital ward
- [ ] Occupational therapy centre
- [ ] Physio and rehabilitation centre
- [ ] Speech therapy centre
- [ ] Other (Please specify) _______________

NOTE : Answer the below question only if answer to Q#2 is General (or peripheral) Neurologist / Neuro Physician OR Neuro-muscular expert

*** 6. Please select the years of experience that you treat the MG patients (Select one option)**

- ○ Less than 5 years
- ○ Between 5 and 10 years
- ○ More than 10 years

NOTE : Answer the below question only if answer to Q#2 is General (or peripheral) Neurologist / Neuro Physician OR Neuro-muscular expert

*** 7. Please select the average number of MG patients you follow on a regular monthly base (Select one option)**

- ○ Less than 5 patients
- ○ Between 5 and 20 patients
- ○ More than 20 patients

NOTE : Display this comment only if answer to Q#2 is General (or peripheral) Neurologist / Neuro Physician OR Neuro-muscular expert

As the first step of our collaboration, we ask you to familiarize with our database Click here to view the overall process flowchart.

Note: The theoretical process of care of MG as it has been defined by the research team was based on the following literature that you could view here.

Thank you for your interest in the participation of the survey.
This survey is intended for General (or peripheral) Neurologist / Neuro Physician or Neuro-muscular expert. Please click Submit

MG Diagnosis

Process analysis

Before answering specific questions please review carefully the key interventions related to diagnosis and assessment process of care [(click here to view the process-flow)]

8. Based on your experience did you find any, not appropriate activity/intervention that was included in the above process flow?

(Select one option)

- ○ Yes
- ○ No

NOTE : Answer the below question only if answer to Q#8 is Yes

9. Could you please flag the not appropriate ones from the following list.

- ☐ Look for Muscle weakness with repetitive muscle use, symmetrically facial, neck, limb, truncal muscles involved or 4 mins of full-arm abduction or 20 mins abductions or speech becomes worse in some pts.
- ☐ Observe for Asymmetrical ptosis, the 5 mins of orbital cooling ice pack test demonstrates bilateral ptosis with >2mm ptosis: Functional ptosis could misdiagnose as MG but in this case eyebrow on the affected eye is lower than the unaffected eyebrow.
- ☐ Check whether MG muscle weakness can be immediately reversed by IV administration of fast-acting acetylcholinesterase inhibitors in patients with observable pareses.
- ☐ Perform Assays: anti-AChR and anti-MuSk anti-bodies.
- ☐ EMG repetitive stimulation.
- ☐ Single-fibre EMG.
- ☐ Acetylcholinesterase inhibitor test.
- ☐ Classify according to MGFA Clinical Classification
- ☐ Measure the outcomes by using instruments such as Quantified Myasthenia Gravis (QMG) Score, MG Quality of Life (MG-QOL 15), MG Manual Muscle Test (MG-MMT).
- ☐ Perform MRI or CT for Thymus screening.

10. Please analyze the below list of activities/interventions included in the subprocess (Diagnosis and assessment) that you examined before.
Could you please rank the importance of those activities from 1 to 10 where 1 is the most important?
(we realize that some activities will get similar importance but to prioritize them, you cannot attribute the same ranking for several activities).
Note: To rank the activities you can either attribute the number directly in the box or drag them in the order you wish.

Look for Muscle weakness with repetitive muscle use, symmetrically facial, neck, limb, truncal muscles involved or 4 mins of full-arm abduction or 20 mins abductions or speech becomes worse in some pts. : _______

Observe for Asymmetrical ptosis, the 5 mins of orbital cooling ice pack test demonstrates bilateral ptosis with >2mm ptosis: Functional ptosis could misdiagnose as MG but in this case eyebrow on the affected eye is lower than the unaffected eyebrow. : _______

Check whether MG muscle weakness can be immediately reversed by IV administration of fast-acting acetylcholinesterase inhibitors in patients with observable pareses. : _______

Perform Assays: anti-AChR and anti-MuSk anti-bodies. : _______

EMG repetitive stimulation. : _______

Single-fibre EMG. : _______

Acetylcholinesterase inhibitor test. : _______

Classify according to MGFA Clinical Classification : _______

Measure the outcomes by using instruments such as Quantified Myasthenia Gravis (QMG) score, MG Quality of Life (MG-QOL 15), MG Manual Muscle Test (MG-MMT). : _______

Perform MRI or CT for Thymus screening. : _______

11. Based on your experience, do you consider the above process flow complete? (Select one option)

- ○ Yes
- ○ No

NOTE : Answer the below question only if answer to Q#11 is No

12. Could you please specify which single most important activity/intervention is missing and include the level of evidence and the eventual reference.

(a) Clinical activity/Intervention

(b) Level of Evidence

(c) Reference/Citation

13. **Based on your practical experience, could you please highlight the single most important potential bottleneck to the above process you may foresee as well at their frequency of occurrence?**
Example: After a hip fracture, clinical guidelines recommend immediate reparative surgery, within 24–48 hours from hospital admission. The organisational prerequisite when implementing this recommendation in real practice is the availability of operating room 24hrs seven days a week. In this case, several bottlenecks can affect this process: for example, during the summertime, it is likely that one hospital could reduce its activities because of the lack of personal for vacation. As a possible consequence, the trauma operating room will be open only five days a week from 8:00-20:00. In such a scenario if a patient is admitted for a hip fracture on Friday afternoon it will be impossible to be compliant with the recommendation. This is how an organisational bottleneck could affect the clinical process.

14. **What is the single most relevant outcome that can be influenced/determined by this part of the process of care? Please, indicate for each outcome its level of evidence (scientific literature and/or clinical expertise and/or your opinion).
Example: After a hip fracture, clinical guidelines recommend immediate reparative surgery, within 24–48 hours from hospital admission. If the surgery is delayed, then there will be an increased risk of negative outcomes (patients death and pressure sores).**

(a) Outcome

(b) Level of Evidence

(c) Reference/Citation

15. Could you please indicate the single most important typology/category of patients that should be excluded from this part of the process of MG care and the determining reason. Please also include the level of evidence of your answer (scientific literature and/or clinical expertise and/or your opinion).
Example: After a hip fracture, clinical guidelines recommend immediate reparative surgery, within 24–48 hours from hospital admission. If the surgery is delayed, then there will be an increase in the risk of death and pressure sores. In this example, the patients with cardiac or renal failure, infections and uncontrolled diabetes or electrolyte abnormalities should be excluded, because they need to be visited cardiologists or nephrologists to evaluate the risk for surgery and often also require additional treatments and tests. This determines the impossibility to respect the appropriate timing of the operating room. Therefore, these patients cannot be included in the pathway.

(a) Typology/category of patients should be excluded

(b) Reasons

(c) Level of evidence

(d) Reference/Citation

16. According to your opinion, what is the most frequent setting where the above process is currently executed?

- ☐ Ambulatory care
- ☐ Hospital day care
- ☐ Hospital in-patient
- ☐ Long-term care (nursing home)
- ☐ Long-term care (rehabilitation)
- ☐ Other (Please specify) _______________

17. How is this list of activities/interventions is differing from the guidelines that you are using in your daily practice?

MG Assessment and
Pharmacological Management

Process analysis

We ask you now to focus on the pharmacological management process of care (click here to view the process-flow) and answer to the questions that will follow.

18. Based on your experience did you find any, not appropriate activity/intervention that was included in the above process flow? (Select one option)

- ○ Yes
- ○ No

NOTE : Answer the below question only if answer to Q#18 is Yes

19. Could you please flag the not appropriate ones from the following list.

- ☐ Patient with mild signs and symptoms: Start with acetylcholinesterase inhibitors.
- ☐ Check whether the patient has Changed in Bowel Pattern – Diarrhea during Acetylcholinesterase inhibitor treatment, if yes then 1.) Maintain a record of intake and output and number of stools. 2.) Monitor electrolyte studies Increase fluid intake. (Note: Carefully monitor Na and water retention in patients taking corticosteroids). 4.) Advance diet from liquid to bland to regular diet as tolerated. 5.) Instruct the patient to avoid foods such as fresh fruit, salads, or spicy or fried foods that may aggravate diarrhoea. 6.) Assess muscle strength to determine changes related to overdosage/under dosage of cholinesterase inhibitor medications. 7.) Instruct the patient to consult a physician for medications to control diarrhoea.
- ☐ If the patient does not respond with acetylcholinesterase inhibitors and the patient has Generalised MG (anti-AChR positive) consider thymectomy (if early onset of thymoma) and prescribe prednisone or prednisone plus azathioprine or mycophenolate mofetil.
- ☐ If the patient does not respond with acetylcholinesterase inhibitors and has Generalised MG (anti-MuSK positive) then prescribe prednisone or prednisone plus azathioprine or mycophenolate mofetil.
- ☐ If the treatment response is not good with prednisone or prednisone plus azathioprine or mycophenolate mofetil then switch to cyclosporin or tacrolimus or methotrexate, IVIg or subcutaneous immunoglobulin
- ☐ If the treatment response is not good with cyclosporin or tacrolimus or methotrexate, IVIg or subcutaneous immunoglobulin then switch to cyclophosphamide, rituximab or eculizumab, serial PE or IVIg
- ☐ If the patient does not respond to cyclophosphamide, rituximab or eculizumab, serial PE or IVIg. Then perform the re-evaluation of diagnosis
- ☐ After the re-evaluation of diagnosis, if the patient evaluated positive to anti-AChR or anti-MuSK then treat by reinstituting last effective dose, high or moderate dose prednisone, PE or IVIg if severe, Add or change immunosuppression.
- ☐ If the treatment response is not good with acetylcholinesterase inhibitors and the patient has only ocular MG then prescribe prednisone. If there is disappearance of the ocular signs and symptoms then continue treatment with lowest dose
- ☐ The patients who take prednisone need to be evaluated: a.) The Alteration in Fluid Balance Related to Na and H2O Retention. b.) Electrolyte Imbalance Related to Potassium Loss. c.) Weight Gain Related to Increased Appetite and Water Retention
- ☐ Patient who take prednisone and evaluated positive to; a.) Alteration in Fluid Balance Related to Na and H2O Retention. b.) Electrolyte Imbalance Related to Potassium Loss. c.) Weight Gain Related to Increased Appetite and Water Retention, then assist the patient with below interventions. 1.) Maintain a record of intake and output. 2.) Daily weights. 3.) Instruct patient on ways to decrease salt intake: – No added salt to food preparation. – No added salt to food at meal time. – Avoid salted snacks. 4.) Read food labels for sodium content. 5.) Avoid canned foods or frozen foods that add salt to food preparation. 6.) Monitor lab values and vital signs, and characteristics of pulse. 7.) Assess changes in muscle strength. 8.) Assist patient to include foods high in potassium, such as citrus fruits, green vegetables, meats, whole grain foods and fish. 9.) Instruct patient on principles of good nutrition. 10.) Instruct patients to avoid fad diets and to consult a physician before starting a weight loss program. 11.)Advise patient that fluctuations in appetite are a normal effect of steroid medications that require a conscious effort to control. 12.) Provide emotional support when changes in body image occur from weight gain from steroids.
- ☐ Patient with severe or moderate oropharyngeal signs/symptoms of MG, or respiratory crisis: Provide Supportive care, Bilevel positive-pressure ventilation, intubate if necessary, stop cholinesterase inhibitors and treat with PE or IVIg

20. Please have a look at the below list of activities/interventions included in the subprocess (MG assessment and pharmacological management) that you examined before. Could you please provide us with your opinion ranking the importance of those activities from 1 to 12 where 1 is the most important?
(we realize that some activities will get similar importance but to prioritize them, you cannot attribute the same ranking for several activities).
Note: To rank the activities you can either attribute the number directly in the box or drag them in the order you wish.

Patient with mild signs and symptoms: Start with acetylcholinesterase inhibitors.	: _______
Check whether the patient has Changed in Bowel Pattern – Diarrhea during Acetylcholinesterase inhibitor treatment, if yes then 1.) Maintain a record of intake and output and number of stools. 2.) Monitor electrolyte studies Increase fluid intake. (Note: Carefully monitor Na and water retention in patients taking corticosteroids). 4.) Advance diet from liquid to bland to regular diet as tolerated. 5.) Instruct the patient to avoid foods such as fresh fruit, salads, or spicy or fried foods that may aggravate diarrhoea. 6.) Assess muscle strength to determine changes related to overdosage/under dosage of cholinesterase inhibitor medications. 7.) Instruct the patient to consult a physician for medications to control diarrhoea.	: _______
If the patient does not respond with acetylcholinesterase inhibitors and the patient has Generalised MG (anti-AChR positive) consider thymectomy (if early onset of thymoma) and prescribe prednisone or prednisone plus azathioprine or mycophenolate mofetil.	: _______
If the patient does not respond with acetylcholinesterase inhibitors and has Generalised MG (anti-MuSK positive) then prescribe prednisone or prednisone plus azathioprine or mycophenolate mofetil.	: _______
If the treatment response is not good with prednisone or prednisone plus azathioprine or mycophenolate mofetil then switch to cyclosporin or tacrolimus or methotrexate, IVIg or subcutaneous immunoglobulin	: _______
If the treatment response is not good with cyclosporin or tacrolimus or methotrexate, IVIg or subcutaneous immunoglobulin then switch to cyclophosphamide, rituximab or eculizumab, serial PE or IVIg	: _______
If the patient does not respond to cyclophosphamide, rituximab or eculizumab, serial PE or IVIg. Then perform the re-evaluation of diagnosis	: _______
After the re-evaluation of diagnosis, if the patient evaluated positive to anti-AChR or anti-MuSK then treat by reinstituting last effective dose, high or moderate dose prednisone, PE or IVIg if severe, Add or change immunosuppression.	: _______
If the treatment response is not good with acetylcholinesterase inhibitors and the patient has only ocular MG then prescribe prednisone. If there is disappearance of the ocular signs and symptoms then continue treatment with lowest dose	: _______
The patients who take prednisone need to be evaluated: a.) The Alteration in Fluid Balance Related to Na and H2O Retention. b.) Electrolyte Imbalance Related to Potassium Loss. c.) Weight Gain Related to Increased Appetite and Water Retention	: _______
Patient who take prednisone and evaluated positive to; a.) Alteration in Fluid Balance Related to Na and H2O Retention. b.) Electrolyte Imbalance Related to Potassium Loss. c.) Weight Gain Related to Increased Appetite and Water Retention, then assist the patient with below interventions. 1.) Maintain a record of intake and output. 2.) Daily weights. 3.) Instruct patient on ways to decrease salt intake: – No added salt to food preparation. – No added salt to food at meal time. – Avoid salted snacks. 4.) Read food labels for sodium content. 5.) Avoid canned foods or frozen foods that add salt to food preparation. 6.) Monitor lab values and vital signs, and characteristics of pulse. 7.) Assess changes in muscle strength. 8.) Assist patient to include foods high in potassium, such as citrus fruits, green vegetables, meats, whole grain foods and fish. 9.) Instruct patient on principles of good nutrition. 10.) Instruct patients to avoid fad diets and to consult a physician before starting a weight loss program. 11.)Advise patient that fluctuations in appetite are a normal effect of steroid medications that require a conscious effort to control. 12.) Provide emotional support when changes in body image occur from weight gain from steroids.	: _______
Patient with severe or moderate oropharyngeal signs/symptoms of MG, or respiratory crisis: Provide Supportive care, Bilevel positive-pressure ventilation, intubate if necessary, stop cholinesterase inhibitors and treat with PE or IVIg	: _______

21. Based on your experience, do you consider the above process flow complete? (Select one option)

- ○ Yes
- ○ No

NOTE : Answer the below question only if answer to Q#21 is No

22. Could you please specify in the below box which activity/intervention is missing (level of evidence and the eventual reference).

(a) clinical activity/Intervention

____

(b) Level of Evidence

____

(c) Reference/Citation

____

23. **Based on your practical experience, could you please highlight the single most important potential bottleneck to the above process you may foresee as well at their frequency of occurrence?**
Example: After a hip fracture, clinical guidelines recommend immediate reparative surgery, within 24–48 hours from hospital admission. The organisational prerequisite when implementing this recommendation in real practice is the availability of operating room 24hrs seven days a week. In this case, several bottlenecks can affect this process: for example during the summertime, it is likely that one hospital could reduce its activities because of the lack of personal for vacation. As a possible consequence, the trauma operating room will be open only five days a week from 8:00-20:00. In such a scenario if a patient is admitted for a hip fracture on Friday afternoon it will be impossible to be compliant with the recommendation. This is how an organisational bottleneck could affect the clinical process.

24. What is the single most relevant outcome that can be influenced/determined by this part of the process of care? Please, indicate for each outcome its level of evidence (scientific literature and/or clinical expertise and/or your opinion). Example: After a hip fracture, clinical guidelines recommend immediate reparative surgery, within 24–48 hours from hospital admission. If the surgery is delayed, then there will be an increased risk of negative outcomes (patients death and pressure sores).

(a) Outcomes

____

(b) Level of evidence

____

(c) Reference/Citation

____

25. Could you please indicate the single most important typology/category of patients that should be excluded from this part of the process of MG care and the determining reason. Please include also the level of evidence of your answer (scientific literature and/or clinical expertise and/or your opinion).
Example: After a hip fracture, clinical guidelines recommend immediate reparative surgery, within 24–48 hours from hospital admission. If the surgery is delayed, then there will be an increase in the risk of death and pressure sores. In this example, the patients with cardiac or renal failure, infections and uncontrolled diabetes or electrolyte abnormalities should be excluded, because they need to be visited cardiologists or nephrologists to evaluate the risk for surgery and often also require additional treatments and tests. This determines the impossibility to respect the appropriate timing of the operating room. Therefore, these patients can not be included in the pathway.

(a) Typology/category of patients should be excluded

____
____
____
____
____

(b) Reasons

____
____
____
____
____

(c) Level of evidence

____
____
____
____
____

(d) Reference/Citation

____
____
____
____
____

26. According to your opinion, what is the most frequent setting where the above process is currently executed?

- ☐ Ambulatory care
- ☐ Hospital day care
- ☐ Hospital in-patient
- ☐ Long-term care (nursing home)
- ☐ Long-term care (rehabilitation)
- ☐ Other (Please specify) _______________

27. How is this list of activities/interventions is differing from the guidelines that you are using in your daily practice?

MG Crisis Assessment and Pharmacological Management

Process analysis

We ask you now to focus on the MG crisis assessment and pharmacological management process of care (click here to view the process-flow) and answer to the questions that will follow.

28. Based on your experience did you find any, not appropriate activity/intervention that was included in the above process flow?

(Select one option)

- ○ Yes
- ○ No

NOTE : Answer the below question only if answer to Q#28 is Yes

29. Could you please flag the not appropriate ones from the following list.

- ☐ Check for bulbar and respiratory weakness
- ☐ Speech and Voice Assessment and Management: a.) If Oral intubation is necessary? - An intubation tube is passing through the pharynx, vocal folds and into the trachea and lungs, use an augmentative communication device. b.) If the patient's inability to communicate it is a failure to wean off the ventilator, necessitating a tracheostomy tube for prolonged ventilator use:- A ventilator Passy-Muir Tracheostomy Speaking Valve (vent valve) Or High-tech augmentative communication device is used.
- ☐ Treat the patients in intensive care with IVIg or plasma exchange plus treatment of infection and other precipitating events. If the patient responds, then Intensify long-term immune suppression.
- ☐ If the patient is not responding to IVIg or plasma exchange then prescribe plasma exchange or IVIg plus Glucocorticoids in high dose, treatment of infection and other precipitating events. If the patient responds, then Intensify long-term immune suppression.
- ☐ If the patient is not responding to plasma exchange or IVIg plus Glucocorticoids in high dose, prescribe other immunosuppressive drugs and treatment of complications. If the patient responds, then Intensify long-term immune suppression.

30. Please have a look at the below list of activities/interventions included in the subprocess (assessment and pharmacological management of MG crisis) that you examined before. Could you please provide us with your opinion ranking the importance of those activities from 1 to 5 where 1 is the most important?
(we realize that some activities will get similar importance but to prioritize them, you cannot attribute the same ranking for several activities).
Note: To rank the activities you can either attribute the number directly in the box or drag them in the order you wish.

Check for bulbar and respiratory weakness	: _______
Speech and Voice Assessment and Management: a.) If Oral intubation is necessary? - An intubation tube is passing through the pharynx, vocal folds and into the trachea and lungs, use an augmentative communication device. b.) If the patient's inability to communicate it is a failure to wean off the ventilator, necessitating a tracheostomy tube for prolonged ventilator use:- A ventilator Passy-Muir Tracheostomy Speaking Valve (vent valve) Or High-tech augmentative communication device is used.	: _______
Treat the patients in intensive care with IVIg or plasma exchange plus treatment of infection and other precipitating events. If the patient responds, then Intensify long-term immune suppression.	: _______
If the patient is not responding to IVIg or plasma exchange then prescribe plasma exchange or IVIg plus Glucocorticoids in high dose, treatment of infection and other precipitating events. If the patient responds, then Intensify long-term immune suppression.	: _______
If the patient is not responding to plasma exchange or IVIg plus Glucocorticoids in high dose, prescribe other immunosuppressive drugs and treatment of complications. If the patient responds, then Intensify long-term immune suppression.	: _______

31. Based on your experience, do you consider the above process flow complete? (Select one option)

- ○ Yes
- ○ No

NOTE : Answer the below question only if answer to Q#31 is No

32. Could you please specify in the below box which activity/intervention is missing (level of evidence and the eventual reference).

(a) clinical activity/Intervention

____

____

____

____

____

(b) Level of Evidence

____

____

____

____

____

(c) Reference/Citation

____

____

____

____

____

33. **Based on your practical experience, could you please highlight the single most important potential bottleneck to the above process you may foresee as well at their frequency of occurrence?**
Example: After a hip fracture, clinical guidelines recommend immediate reparative surgery, within 24–48 hours from hospital admission. The organisational prerequisite when implementing this recommendation in real practice is the availability of operating room 24hrs seven days a week. In this case, several bottlenecks can affect this process: for example during the summertime, it is likely that one hospital could reduce its activities because of the lack of personal for vacation. As a possible consequence, the trauma operating room will be open only five days a week from 8:00-20:00. In such a scenario if a patient is admitted for a hip fracture on Friday afternoon it will be impossible to be compliant with the recommendation. This is how an organisational bottleneck could affect the clinical process.

34. What is the single most relevant outcome that can be influenced/determined by this part of the process of care? Please, indicate for each outcome its level of evidence (scientific literature and/or clinical expertise and/or your opinion). Example: After a hip fracture, clinical guidelines recommend immediate reparative surgery, within 24–48 hours from hospital admission. If the surgery is delayed, then there will be an increased risk of negative outcomes (patients death and pressure sores).

(a) Outcomes

_____
_____
_____
_____
_____

(b) Level of evidence

_____
_____
_____
_____
_____

(c) Reference/Citation

_____
_____
_____
_____
_____

35. Could you please indicate the single most important typology/category of patients that should be excluded from this part of the process of MG care and the determining reason. Please include also the level of evidence of your answer (scientific literature and/or clinical expertise and/or your opinion).
Example: After a hip fracture, clinical guidelines recommend immediate reparative surgery, within 24–48 hours from hospital admission. If the surgery is delayed, then there will be an increase in the risk of death and pressure sores. In this example, the patients with cardiac or renal failure, infections and uncontrolled diabetes or electrolyte abnormalities should be excluded, because they need to be visited cardiologists or nephrologists to evaluate the risk for surgery and often also require additional treatments and tests. This determines the impossibility to respect the appropriate timing of the operating room. Therefore, these patients can not be included in the pathway.

(a) Typology/category of patients should be excluded

____
____
____
____
____

(b) Reasons

____
____
____
____
____

(c) Level of evidence

____
____
____
____
____

(d) Reference/Citation

____
____
____
____
____

36. According to your opinion, what is the most frequent setting where the above process is currently executed?

- ☐ Ambulatory care
- ☐ Hospital day care
- ☐ Hospital in-patient
- ☐ Long-term care (nursing home)
- ☐ Long-term care (rehabilitation)
- ☐ Other (Please specify) _______________

37. How is this list of activities/interventions is differing from the guidelines that you are using in your daily practice?

Process Analysis

We ask you now to focus on the assessment and management of speech, swallowing and dental issues process of care [(click here to view the process-flow)] and answer to the questions that will follow.

38. Based on your experience did you find any, not appropriate activity/intervention that was included in the above process flow? (Select one option)

- ○ Yes
- ○ No

NOTE : Answer the below question only if answer to Q#38 is Yes

39. Could you please flag the not appropriate ones from the following list.

- ☐ Clinical swallowing examination: A.) Evaluate the musculature functioning of cranial nerves V, VII, IX, X, XI and XII is needed in all individuals with MG, especially patients with bulbar manifestations. B.)Test the fatigability of the facial, lingual and laryngeal functioning.
- ☐ Instrumental swallowing examination by using a Modified Barium swallow study (MBS)/ Fiberoptic Endoscopic evaluation of swallowing (FEES): A.) Evaluation of oropharyngeal and pharyngeal dysphagia. B.)View the hyolaryngeal elevation, tongue base to posterior pharyngeal wall approximation, pharyngeal contraction, upper esophageal sphincter (UES) opening and to perform a screen of esophageal motility by using MBS. C.) View the laryngeal physiology by using FEES. D.) Visualization of Pharyngeal Physiology. E.) Fatigability of Pharyngeal Functioning.
- ☐ Swallowing Rehabilitation: For improving one's oropharyngeal muscular baseline strength may decrease the degree of dysphagia.
- ☐ Lifetime Swallowing Plan: A.) Every three months assess swallowing in order to manage potential aspiration before it contributes to pulmonary complication and possible myasthenia crisis. B.) The speech-language pathologist (SLP) should make sure that patients and their caregivers educated to the signs and symptoms of dysphagia and aspiration. They should be knowledgeable and able to detect wet voice, throat clearing, coughing, increased chest congestion after eating, etc. C.) Patients should be instructed to periodically assess temperature and be alert to indicators of pneumonia.
- ☐ Compensatory Maneuvers: chin tuck, head turn or supraglottic swallow for eliminating or decreasing aspiration.
- ☐ Modify dental treatment to accommodate altered muscle strength: A.) Oral hygiene aids: 1.) Use Electric toothbrush. 2.) Use Manual toothbrush with a modified handle for ease of grasp. B.) Mouth prop, rubber dam isolation and saliva ejector use during dental treatment: 1.) Mouth props prevent muscle strain of having to hold the mouth open during treatment. 2.) Rubber dam isolation prevents worries about choking. By keeping dental materials and water spray out of the throat 3.) Saliva ejector suction tubing may be held and controlled by the patient to help avoid drooling and choking on saliva during procedures. C.) Dental chair position during Dental treatment: It is usually done in a reclining position. Let the dentist know if the patient is so far back that he feels like the throat is closing off. D.) Scheduled rest periods: Let the dentist know if the patient will need frequent rest breaks during treatment. E.) Compromised ability to manage complete dentures: 1.) The inability of the flaccid muscles to assist in retaining the lower denture and to maintain a peripheral seal for the upper denture. 2.) Overextended and over contoured maxillary dentures with thick flanges that impinge upon muscle and frenal attachments can lead to muscle fatigue and altered salivation. 3.) Ill-fitting dentures may exacerbate symptoms of difficulty in closing the mouth, tongue fatigue, a tight upper lip, dry mouth, impaired phonation, dysphagia and masticatory problems. F.) Monitor oral side effects/drug interactions of drugs/ therapies used to treat MG.
- ☐ Other (Please specify) ________________

40. Please have a look at the below list of activities/interventions included in the subprocess (Speech, swallowing and dental functioning) that you examined before. Could you please provide us with your opinion ranking the importance of those activities from 1 to 6 where 1 is the most important?
(we realize that some activities will get similar importance but to prioritize them, you cannot attribute the same ranking for several activities).
Note: To rank the activities you can either attribute the number directly in the box or drag them in the order you wish.

Clinical swallowing examination: A.) Evaluate the musculature functioning of cranial nerves V, VII, IX, X, XI and XII is needed in all individuals with MG, especially patients with bulbar manifestations. B.)Test the fatigability of the facial, lingual and laryngeal functioning.	: _______
Instrumental swallowing examination by using a Modified Barium swallow study (MBS)/ Fiberoptic Endoscopic evaluation of swallowing (FEES): A.) Evaluation of oropharyngeal and pharyngeal dysphagia. B.)View the hyolaryngeal elevation, tongue base to posterior pharyngeal wall approximation, pharyngeal contraction, upper esophageal sphincter (UES) opening and to perform a screen of esophageal motility by using MBS. C.) View the laryngeal physiology by using FEES. D.) Visualization of Pharyngeal Physiology. E.) Fatigability of Pharyngeal Functioning.	: _______
Swallowing Rehabilitation: For improving one's oropharyngeal muscular baseline strength may decrease the degree of dysphagia.	: _______
Lifetime Swallowing Plan: A.) Every three months assess swallowing in order to manage potential aspiration before it contributes to pulmonary complication and possible myasthenia crisis. B.) The speech-language pathologist (SLP) should make sure that patients and their caregivers educated to the signs and symptoms of dysphagia and aspiration. They should be knowledgeable and able to detect wet voice, throat clearing, coughing, increased chest congestion after eating, etc. C.) Patients should be instructed to periodically assess temperature and be alert to indicators of pneumonia.	: _______
Compensatory Maneuvers: chin tuck, head turn or supraglottic swallow for eliminating or decreasing aspiration.	: _______
Modify dental treatment to accommodate altered muscle strength: A.) Oral hygiene aids: 1.) Use Electric toothbrush. 2.) Use Manual toothbrush with a modified handle for ease of grasp. B.) Mouth prop, rubber dam isolation and saliva ejector use during dental treatment: 1.) Mouth props prevent muscle strain of having to hold the mouth open during treatment. 2.) Rubber dam isolation prevents worries about choking. By keeping dental materials and water spray out of the throat 3.) Saliva ejector suction tubing may be held and controlled by the patient to help avoid drooling and choking on saliva during procedures. C.) Dental chair position during Dental treatment: It is usually done in a reclining position. Let the dentist know if the patient is so far back that he feels like the throat is closing off. D.) Scheduled rest periods: Let the dentist know if the patient will need frequent rest breaks during treatment. E.) Compromised ability to manage complete dentures: 1.) The inability of the flaccid muscles to assist in retaining the lower denture and to maintain a peripheral seal for the upper denture. 2.) Overextended and over contoured maxillary dentures with thick flanges that impinge upon muscle and frenal attachments can lead to muscle fatigue and altered salivation. 3.) Ill-fitting dentures may exacerbate symptoms of difficulty in closing the mouth, tongue fatigue, a tight upper lip, dry mouth, impaired phonation, dysphagia and masticatory problems. F.) Monitor oral side effects/drug interactions of drugs/ therapies used to treat MG.	: _______

41. Based on your experience, do you consider the above process flow complete?

(Select one option)

- ◯ Yes
- ◯ No

NOTE : Answer the below question only if answer to Q#41 is No

42. Could you please specify in the below box which activity/intervention is missing (level of evidence and the eventual reference).

(a) clinical activity/Intervention

____

____

____

____

____

(b) Level of Evidence

____

____

____

____

____

(c) Reference/Citation

____

____

____

____

____

43. Based on your practical experience, could you please highlight the single most important potential bottleneck to the above process you may foresee as well at their frequency of occurrence?
Example: After a hip fracture, clinical guidelines recommend immediate reparative surgery, within 24–48 hours from hospital admission. The organisational prerequisite when implementing this recommendation in real practice is the availability of operating room 24hrs seven days a week. In this case, several bottlenecks can affect this process: for example during the summertime, it is likely that one hospital could reduce its activities because of the lack of personal for vacation. As a possible consequence, the trauma operating room will be open only five days a week from 8:00-20:00. In such a scenario if a patient is admitted for a hip fracture on Friday afternoon it will be impossible to be compliant with the recommendation. This is how an organisational bottleneck could affect the clinical process.

44. What is the single most relevant outcome that can be influenced/determined by this part of the process of care? Please, indicate for each outcome its level of evidence (scientific literature and/or clinical expertise and/or your opinion). Example: After a hip fracture, clinical guidelines recommend immediate reparative surgery, within 24–48 hours from hospital admission. If the surgery is delayed, then there will be an increased risk of negative outcomes (patients death and pressure sores).

(a) Outcomes

(b) Level of evidence

(c) Reference/Citation

45. Could you please indicate the single most important typology/category of patients that should be excluded from this part of the process of MG care and the determining reason. Please include also the level of evidence of your answer (scientific literature and/or clinical expertise and/or your opinion).
Example: After a hip fracture, clinical guidelines recommend immediate reparative surgery, within 24–48 hours from hospital admission. If the surgery is delayed, then there will be an increase in the risk of death and pressure sores. In this example, the patients with cardiac or renal failure, infections and uncontrolled diabetes or electrolyte abnormalities should be excluded, because they need to be visited cardiologists or nephrologists to evaluate the risk for surgery and often also require additional treatments and tests. This determines the impossibility to respect the appropriate timing of the operating room. Therefore, these patients can not be included in the pathway.

(a) Typology/category of patients should be excluded

____

(b) Reasons

____

(c) Level of evidence

____

(d) Reference/Citation

____

46. According to your opinion, what is the most frequent setting where the above process is currently executed?

- ☐ Ambulatory care
- ☐ Hospital day care
- ☐ Hospital in-patient
- ☐ Long-term care (nursing home)
- ☐ Long-term care (rehabilitation)
- ☐ Other (Please specify) _______________

47. How is this list of activities/interventions is differing from the guidelines that you are using in your daily practice?

Process Analysis

We ask you now to focus on the assessment and management of occupational, physical, and respiratory issues process of care (click here to view the process-flow) and answer to the questions that will follow.

48. Based on your experience did you find any, not appropriate activity/intervention that was included in the above process flow? (Select one option)

- ○ Yes
- ○ No

NOTE : Answer the below question only if answer to Q#48 is Yes

49. Could you please flag the not appropriate ones from the following list.

- ☐ Activities of Daily Living: 1.) Functional Independence Measures by using a.) Barthel Index. b.) Klein-Bell. 2.) Canadian Occupational Performance Measure. 3.) Myasthenia Gravis Activities of Daily Living Profile
- ☐ Strength: Measure the Grip strength using dynamometry.
- ☐ Fatigue: a.) Perceived Exertion Scale b.) Modified Fatigue Scale.
- ☐ Vision/Ocular Motor Function: 1.) Extra Ocular Motility Exam. 2.) Hirschberg Light Reflex test. 3.) Maddox Rod test. 4.) Ptosis measurement in millimeters.
- ☐ Instrumental Activities of Daily Living : 1.) Assessment of Motor and Process Skills. 2.) Canadian Occupational Performance Measure
- ☐ Assessment of respiratory function: 1.) Check whether the patient is able to lie flat or bend over without shortness of breath 2.) Assess chest expansion and auscultate the chest in all quadrants for air entry or crackles. 3.) Observe for shortness of breath, increased respiratory effort or frequent inspiratory gasps. 4.) check for the rate, rhythm, and quality of respiration's and the presence of anxiety or restlessness. 5.) Oxygen saturation and blood gas analysis (additional).
- ☐ Basic Self-Care: 1.) Use bathroom durable medical equipment to allow one to shower and groom while seated. 2.) Organize supplies and clothing such that minimal energy is spent obtaining items.
- ☐ Home Management: 1.) Perform laundry in smaller loads. 2.) Use laundry hampers on casters that allow for ease in rolling rather than lifting. 3.) Organize frequently used cooking equipment/ utensils to allow ease in reaching and reduced trips to collect. 4.) Use lightweight equipment rather than heavy vacuum cleaners or mops.
- ☐ Shopping: 1.) Plan shopping trips during the time of day that one has the most energy. 2.) Consider grocery stores that provide a "call-in" and pick-up service.
- ☐ Parenting: Arrange diaper changing stations to allow performance while seated.
- ☐ Care of Pets: 1.) Use elevated food container on casters. 2.) Use elevated feeding stations. 3.) Hire someone to take pets for a walk.
- ☐ Leisure Activities: 1.) Perform more strenuous activities during peak medication response. 2.) Learn early signs of fatigue and allow sufficient recovery time. 3.) Balance strenuous activities with ones that require less physical exertion.
- ☐ Sexual Activity: 1.) Plan sexual activities at times when concern for fatigue is not a factor. 2.) Utilize positions that require less energy for posture.

50. Please have a look at the below list of activities/interventions included in the subprocess (Occupational, Physical and Respiratory assessment and management) that you examined before. Could you please provide us with your opinion ranking the importance of those activities from 1 to 13 where 1 is the most important?
(we realize that some activities will get similar importance but to prioritize them, you cannot attribute the same ranking for several activities).
Note: To rank the activities you can either attribute the number directly in the box or drag them in the order you wish.

Activities of Daily Living: 1.) Functional Independence Measures by using a.) Barthel Index. b.) Klein-Bell. 2.) Canadian Occupational Performance Measure. 3.) Myasthenia Gravis Activities of Daily Living Profile : _______

Strength: Measure the Grip strength using dynamometry. : _______

Fatigue: a.) Perceived Exertion Scale b.) Modified Fatigue Scale. : _______

Vision/Ocular Motor Function: 1.) Extra Ocular Motility Exam. 2.) Hirschberg Light Reflex test. 3.) Maddox Rod test. 4.) Ptosis measurement in millimeters. : _______

Instrumental Activities of Daily Living : 1.) Assessment of Motor and Process Skills. 2.) Canadian Occupational Performance Measure : _______

Assessment of respiratory function: 1.) Check whether the patient is able to lie flat or bend over without shortness of breath 2.) Assess chest expansion and auscultate the chest in all quadrants for air entry or crackles. 3.) Observe for shortness of breath, increased respiratory effort or frequent inspiratory gasps. 4.) check for the rate, rhythm, and quality of respiration's and the presence of anxiety or restlessness. 5.) Oxygen saturation and blood gas analysis (additional). : _______

Basic Self-Care: 1.) Use bathroom durable medical equipment to allow one to shower and groom while seated. 2.) Organize supplies and clothing such that minimal energy is spent obtaining items. : _______

Home Management: 1.) Perform laundry in smaller loads. 2.) Use laundry hampers on casters that allow for ease in rolling rather than lifting. 3.) Organize frequently used cooking equipment/ utensils to allow ease in reaching and reduced trips to collect. 4.) Use lightweight equipment rather than heavy vacuum cleaners or mops. : _______

Shopping: 1.) Plan shopping trips during the time of day that one has the most energy. 2.) Consider grocery stores that provide a "call-in" and pick-up service. : _______

Parenting: Arrange diaper changing stations to allow performance while seated. : _______

Care of Pets: 1.) Use elevated food container on casters. 2.) Use elevated feeding stations. 3.) Hire someone to take pets for a walk. : _______

Leisure Activities: 1.) Perform more strenuous activities during peak medication response. 2.) Learn early signs of fatigue and allow sufficient recovery time. 3.) Balance strenuous activities with ones that require less physical exertion. : _______

Sexual Activity: 1.) Plan sexual activities at times when concern for fatigue is not a factor. 2.) Utilize positions that require less energy for posture. : _______

51. Based on your experience, do you consider the above process flow complete? (Select one option)

○ Yes

○ No

NOTE : Answer the below question only if answer to Q#51 is No

52. Could you please specify in the below box which activity/intervention is missing (level of evidence and the eventual reference).

(a) Clinical activity/Intervention

(b) Level of evidence

(c) Reference

53. **Based on your practical experience, could you please highlight the single most important potential bottleneck to the above process you may foresee as well at their frequency of occurrence?**
Example: After a hip fracture, clinical guidelines recommend immediate reparative surgery, within 24–48 hours from hospital admission. The organisational prerequisite when implementing this recommendation in real practice is the availability of operating room 24hrs seven days a week. In this case, several bottlenecks can affect this process: for example during the summertime, it is likely that one hospital could reduce its activities because of the lack of personal for vacation. As a possible consequence, the trauma operating room will be open only five days a week from 8:00-20:00. In such a scenario if a patient is admitted for a hip fracture on Friday afternoon it will be impossible to be compliant with the recommendation. This is how an organisational bottleneck could affect the clinical process.

54. What is the single most relevant outcome that can be influenced/determined by this part of the process of care? Please, indicate for each outcome its level of evidence (scientific literature and/or clinical expertise and/or your opinion). Example: After a hip fracture, clinical guidelines recommend immediate reparative surgery, within 24–48 hours from hospital admission. If the surgery is delayed, then there will be an increased risk of negative outcomes (patients death and pressure sores).

(a) Outcomes

(b) Level of evidence

(c) Reference/Citation

55. Could you please indicate the single most important typology/category of patients that should be excluded from this part of the process of MG care and the determining reason. Please include also the level of evidence of your answer (scientific literature and/or clinical expertise and/or your opinion).
Example: After a hip fracture, clinical guidelines recommend immediate reparative surgery, within 24–48 hours from hospital admission. If the surgery is delayed, then there will be an increase in the risk of death and pressure sores. In this example, the patients with cardiac or renal failure, infections and uncontrolled diabetes or electrolyte abnormalities should be excluded, because they need to be visited cardiologists or nephrologists to evaluate the risk for surgery and often also require additional treatments and tests. This determines the impossibility to respect the appropriate timing of the operating room. Therefore, these patients can not be included in the pathway.

(a) Typology/category of patients should be excluded

____

____

____

____

____

(b) Reasons

____

____

____

____

____

(c) Level of evidence

____

____

____

____

____

(d) Reference/Citation

____

____

____

____

____

56. According to your opinion, what is the most frequent setting where the above process is currently executed?

- ☐ Ambulatory care
- ☐ Hospital day care
- ☐ Hospital in-patient
- ☐ Long-term care (nursing home)
- ☐ Long-term care (rehabilitation)
- ☐ Other (Please specify) _______________

57. How is this list of activities/interventions is differing from the guidelines that you are using in your daily practice?

Psychological Factors
Assessment and Management

Process analysis

We ask you now to focus on the psychological factors assessment and management process of care (click here to view the process-flow) and answer to the questions that will follow.

58. Based on your experience did you find any, not appropriate activity/intervention that was included in the above process flow? (Select one option)

- ◯ Yes
- ◯ No

NOTE : Answer the below question only if answer to Q#58 is Yes

59. Could you please flag the not appropriate ones from the following list.

- ☐ Assessment of Potentially dysfunctional self-concepts.
- ☐ Assessment of Ineffective Coping Mechanisms.
- ☐ Assessment of Altered role performance.
- ☐ Assessment of Anxiety-related to the disease process and lifestyle alterations, Fear of death: patient family and friends.
- ☐ Interventions for Potentially dysfunctional self-concepts: 1.) Encourage patients to verbalize the meaning of the illness/loss (i.e., "How do you feel about what is happening to you?") 2.) Listen attentively and compassionately. 3.) Since appearances may greatly alter and weakness may leave patients unable to take care of grooming needs, help them to look their best. 4.) Be honest about the realities of the illness; encourage patients to seek help if denial becomes detrimental. 5.) Facilitate acceptance; help patients set realistic, short-term goals so that success may be achieved. 6.) Encourage patients to do the things that they are capable of doing. 7.) Share hopeful aspects of the disease with patients and family. 8.) Recognize that the family too will be experiencing grief for the loss of the way the patient "used to be."
- ☐ Interventions for Ineffective coping mechanism: 1.) Help patients to identify how they coped with adversity in the past
- ☐ Interventions for Role performance strategies: 1.) Determine what their usual coping mechanisms are, and how they can best be used to cope with the MG. 2.) Assist patients in identifying factors in their environment that have the potential to undermine positive adaptation. 3.) Involve patients in planning and decision making regarding care. 4.) Give patients and family information regarding the disease, medications, emergency measures, and precautions for living with MG after discharge from the acute care setting. 5.) Explore patient role changes so that they will be less threatening 6.) Supply information on local MG chapter. Relationships can be formed with others with the disease and be a great source of strength to patients and family
- ☐ Interventions for Anxiety-related to the disease process and Lifestyle alterations, Fear of death: patient family and friends 1.) Active Listening. 2.) Refer if necessary for counselling or therapy to deal with anxiety.

60. Please have a look at the below list of activities/interventions included in the subprocess (Psychological factors assessment and management) that you examined before. Could you please provide us with your opinion ranking the importance of those activities from 1 to 8 where 1 is the most important?
(we realize that some activities will get similar importance but to prioritize them, you cannot attribute the same ranking for several activities).
Note: To rank the activities you can either attribute the number directly in the box or drag them in the order you wish.

Assessment of Potentially dysfunctional self-concepts.	: _______
Assessment of Ineffective Coping Mechanisms.	: _______
Assessment of Altered role performance.	: _______
Assessment of Anxiety-related to the disease process and lifestyle alterations, Fear of death: patient family and friends.	: _______
Interventions for Potentially dysfunctional self-concepts: 1.) Encourage patients to verbalize the meaning of the illness/loss (i.e., “How do you feel about what is happening to you?”) 2.) Listen attentively and compassionately. 3.) Since appearances may greatly alter and weakness may leave patients unable to take care of grooming needs, help them to look their best. 4.) Be honest about the realities of the illness; encourage patients to seek help if denial becomes detrimental. 5.) Facilitate acceptance; help patients set realistic, short-term goals so that success may be achieved. 6.) Encourage patients to do the things that they are capable of doing. 7.) Share hopeful aspects of the disease with patients and family. 8.) Recognize that the family too will be experiencing grief for the loss of the way the patient “used to be.”	: _______
Interventions for Ineffective coping mechanism: 1.) Help patients to identify how they coped with adversity in the past	: _______
Interventions for Role performance strategies: 1.) Determine what their usual coping mechanisms are, and how they can best be used to cope with the MG. 2.) Assist patients in identifying factors in their environment that have the potential to undermine positive adaptation. 3.) Involve patients in planning and decision making regarding care. 4.) Give patients and family information regarding the disease, medications, emergency measures, and precautions for living with MG after discharge from the acute care setting. 5.) Explore patient role changes so that they will be less threatening 6.) Supply information on local MG chapter. Relationships can be formed with others with the disease and be a great source of strength to patients and family	: _______
Interventions for Anxiety-related to the disease process and Lifestyle alterations, Fear of death: patient family and friends 1.) Active Listening. 2.) Refer if necessary for counselling or therapy to deal with anxiety.	: _______

61. Based on your experience, do you consider the above process flow complete? (Select one option)

- ○ Yes
- ○ No

NOTE : Answer the below question only if answer to Q#61 is No

62. Could you please specify in the below box which activity/intervention is missing (level of evidence and the eventual reference).

(a) clinical activity/Intervention

(b) Level of Evidence

(c) Reference/Citation

63. **Based on your practical experience, could you please highlight the single most important potential bottleneck to the above process you may foresee as well at their frequency of occurrence?**
Example: After a hip fracture, clinical guidelines recommend immediate reparative surgery, within 24–48 hours from hospital admission. The organisational prerequisite when implementing this recommendation in real practice is the availability of operating room 24hrs seven days a week. In this case, several bottlenecks can affect this process: for example during the summertime, it is likely that one hospital could reduce its activities because of the lack of personal for vacation. As a possible consequence, the trauma operating room will be open only five days a week from 8:00-20:00. In such a scenario if a patient is admitted for a hip fracture on Friday afternoon it will be impossible to be compliant with the recommendation. This is how an organisational bottleneck could affect the clinical process.

64. What is the single most relevant outcome that can be influenced/determined by this part of the process of care? Please, indicate for each outcome its level of evidence (scientific literature and/or clinical expertise and/or your opinion). Example: After a hip fracture, clinical guidelines recommend immediate reparative surgery, within 24–48 hours from hospital admission. If the surgery is delayed, then there will be an increased risk of negative outcomes (patients death and pressure sores).

(a) Outcomes

(b) Level of evidence

(c) Reference/Citation

65. Could you please indicate the single most important typology/category of patients that should be excluded from this part of the process of MG care and the determining reason. Please include also the level of evidence of your answer (scientific literature and/or clinical expertise and/or your opinion).
Example: After a hip fracture, clinical guidelines recommend immediate reparative surgery, within 24–48 hours from hospital admission. If the surgery is delayed, then there will be an increase in the risk of death and pressure sores. In this example, the patients with cardiac or renal failure, infections and uncontrolled diabetes or electrolyte abnormalities should be excluded, because they need to be visited cardiologists or nephrologists to evaluate the risk for surgery and often also require additional treatments and tests. This determines the impossibility to respect the appropriate timing of the operating room. Therefore, these patients can not be included in the pathway.

(a) Typology/category of patients should be excluded

(b) Reasons

(c) Level of evidence

(d) Reference/Citation

66. According to your opinion, what is the most frequent setting where the above process is currently executed?

☐ Ambulatory care
☐ Hospital day care
☐ Hospital in-patient
☐ Long-term care (nursing home)
☐ Long-term care (rehabilitation)
☐ Other (Please specify) _______________

67. How is this list of activities/interventions is differing from the guidelines that you are using in your daily practice?

Life-style Factors
Assessment and Management

Process analysis

We ask you now to focus on the life-style factors assessment and management process of care (click here to view the process-flow) and answer to the questions that will follow.

68. Based on your experience did you find any, not appropriate activity/intervention that was included in the above process flow? (Select one option)

- ○ Yes
- ○ No

NOTE : Answer the below question only if answer to Q#68 is Yes

69. Could you please flag the not appropriate ones from the following list.

- ☐ Nutritional assessment
- ☐ Stress and Coping Skills assessment
- ☐ Infection Control and Health Maintenance assessment
- ☐ Interventions for good Nutrition: 1.) Weight Control-low fat foods, lean protein, good carbohydrates and caloric intake based on age, gender, height, weight and activity level. 2.) Low Sodium -to reduce edema, breathing and other health problems. 3.) Potassium intake (bananas, avocado, broccoli, potato, citrus fruits, fish, dairy). Potassium depletion can occur due to corticosteroid treatment. 4.) Calcium (dairy, green leafy vegetables, broccoli, cauliflower, egg yolks, lentils, soft bones of salmon and sardines) for strong bones and teeth.
- ☐ Interventions for stress management and coping Skills: 1.) Avoid a sedentary lifestyle. 2.) Progressive relaxation, yoga, deep-breathing, physical activity (walking, stationary ergometer, weight training, treadmill, swimming), visualization, adequate rest and proper nutrition. 4.) Support groups, stress management programs, socialization and religious/spiritual activities. 5.) Psychological counselling.
- ☐ Interventions for infection control and health maintenance: 1.) Proper handwashing (20 seconds with soap and water, dry well, lotion on dry skin). 2.) Maintain good dental care and oral hygiene. 3.) Avoid large crowds and individuals with upper respiratory infections or other contagious illnesses. 4.) Keep current on immunizations as advised by the health care provider.

70. Please have a look at the below list of activities/interventions included in the subprocess (Life-style factors assessment and management) that you examined before. Could you please provide us with your opinion ranking the importance of those activities from 1 to 6 where 1 is the most important?
(we realize that some activities will get similar importance but to prioritize them, you cannot attribute the same ranking for several activities).
Note: To rank the activities you can either attribute the number directly in the box or drag them in the order you wish.

Nutritional assessment : ______

Stress and Coping Skills assessment : ______

Infection Control and Health Maintenance assessment : ______

Interventions for good Nutrition: 1.) Weight Control-low fat foods, lean protein, good carbohydrates and caloric intake based on age, gender, height, weight and activity level. 2.) Low Sodium -to reduce edema, breathing and other health problems. 3.) Potassium intake (bananas, avocado, broccoli, potato, citrus fruits, fish, dairy). Potassium depletion can occur due to corticosteroid treatment. 4.) Calcium (dairy, green leafy vegetables, broccoli, cauliflower, egg yolks, lentils, soft bones of salmon and sardines) for strong bones and teeth. : ______

Interventions for stress management and coping Skills: 1.) Avoid a sedentary lifestyle. 2.) Progressive relaxation, yoga, deep-breathing, physical activity (walking, stationary ergometer, weight training, treadmill, swimming), visualization, adequate rest and proper nutrition. 4.) Support groups, stress management programs, socialization and religious/spiritual activities. 5.) Psychological counselling. : ______

Interventions for infection control and health maintenance: 1.) Proper handwashing (20 seconds with soap and water, dry well, lotion on dry skin). 2.) Maintain good dental care and oral hygiene. 3.) Avoid large crowds and individuals with upper respiratory infections or other contagious illnesses. 4.) Keep current on immunizations as advised by the health care provider. : ______

71. Based on your experience, do you consider the above process flow complete? (Select one option)

- ○ Yes
- ○ No

NOTE : Answer the below question only if answer to Q#71 is No

72. Could you please specify in the below box which activity/intervention is missing (level of evidence and the eventual reference).

(a) clinical activity/Intervention

____

(b) Level of Evidence

____

(c) Reference/Citation

____

73. Based on your practical experience, could you please highlight the single most important potential bottleneck to the above process you may foresee as well at their frequency of occurrence?
Example: After a hip fracture, clinical guidelines recommend immediate reparative surgery, within 24–48 hours from hospital admission. The organisational prerequisite when implementing this recommendation in real practice is the availability of operating room 24hrs seven days a week. In this case, several bottlenecks can affect this process: for example during the summertime, it is likely that one hospital could reduce its activities because of the lack of personal for vacation. As a possible consequence, the trauma operating room will be open only five days a week from 8:00-20:00. In such a scenario if a patient is admitted for a hip fracture on Friday afternoon it will be impossible to be compliant with the recommendation. This is how an organisational bottleneck could affect the clinical process.

74. What is the single most relevant outcome that can be influenced/determined by this part of the process of care? Please, indicate for each outcome its level of evidence (scientific literature and/or clinical expertise and/or your opinion). Example: After a hip fracture, clinical guidelines recommend immediate reparative surgery, within 24–48 hours from hospital admission. If the surgery is delayed, then there will be an increased risk of negative outcomes (patients death and pressure sores).

(a) Outcomes

(b) Level of evidence

(c) Reference/Citation

75. Could you please indicate the single most important typology/category of patients that should be excluded from this part of the process of MG care and the determining reason. Please include also the level of evidence of your answer (scientific literature and/or clinical expertise and/or your opinion).
Example: After a hip fracture, clinical guidelines recommend immediate reparative surgery, within 24–48 hours from hospital admission. If the surgery is delayed, then there will be an increase in the risk of death and pressure sores. In this example, the patients with cardiac or renal failure, infections and uncontrolled diabetes or electrolyte abnormalities should be excluded, because they need to be visited cardiologists or nephrologists to evaluate the risk for surgery and often also require additional treatments and tests. This determines the impossibility to respect the appropriate timing of the operating room. Therefore, these patients can not be included in the pathway.

(a) Typology/category of patients should be excluded

(b) Reasons

(c) Level of evidence

(d) Reference/Citation

76. According to your opinion, what is the most frequent setting where the above process is currently executed?

- ☐ Ambulatory care
- ☐ Hospital day care
- ☐ Hospital in-patient
- ☐ Long-term care (nursing home)
- ☐ Long-term care (rehabilitation)
- ☐ Other (Please specify) _______________

77. How is this list of activities/interventions is differing from the guidelines that you are using in your daily practice?
