# Supplementary material for: Development of a Model Care Pathway for Myasthenia Gravis
Source: Int J Environ Res Public Health. 2021 Nov 4;18(21):11591. doi: 10.3390/ijerph182111591 (PMC8582978; doi:10.3390/ijerph182111591)
Supplement: Supplementary file 1 [file ijerph-18-11591-s001.zip › S3_Characteristics of included guidlines.pdf]

S3 - Characteristics of included guidelines across the USA, Europe, Japan, and their overall quality scores by AGREE-II tool.

N	Title	Year	Society/Developer	Country	Developed methods	AGREE-II. Overall Quality
**1**	International Consensus Guidance for the Management of Myasthenia Gravis	2016	American Academy of Neurology	America	Consensus-based	5/7
**2**	Plasmapheresis in neurologic disorders	2011	American Academy of Neurology	America	Evidence-based	6/7
**3**	Intravenous immunoglobulin in the treatment of neuromuscular disorders	2012	American Academy of Neurology	America	Evidence-based	6/7
**4**	Repetitive nerve stimulation and single-fiber electromyography in the evaluation of patients with suspected myasthenia gravis or Lambert-Eaton myasthenic syndrome	2015	American Association of Electrodiagnostic Medicine	America	Literature review	5/7
**5**	Thymectomy for autoimmune myasthenia gravis	2000	American Academy of Neurology	America	Evidence-based review	6/7
**6**	Care of the Patient with Myasthenia Gravis	2014	American Association of Neuroscience Nurses	America	Evidence-based review	4/7
**7**	EFNS/ENS Guidelines for the treatment of ocular myasthenia	2013	European Federation of Neurological Societies	Europe	Literature review	6/7
**8**	Guidelines for treatment of autoimmune neuromuscular transmission disorders	2010	European Federation of Neurological Societies	Europe	Literature review	6/7
**9**	EFNS guidelines for the use of intravenous immunoglobulin in treatment of neurological diseases	2008	European Federation of Neurological Societies	Europe	Literature review	5/7
**10**	Myasthenia gravis: Association of British Neurologists' management guidelines	2015	Association of British Neurologists	UK	Literature review	6/7
**11**	Myasthenia in pregnancy: best practice guidelines from a UK multispecialty working group.	2013	UK multispecialty working group	UK	Literature review	6/7
**12**	Consensus statement: the use of intravenous immunoglobulin in the treatment of neuromuscular conditions.	2009	American Association of Neuromuscular & Electrodiagnostic Medicine	America	Consensus-based	4/7
**13**	Japanese clinical guidelines for myasthenia gravis: Putting into practice	2015	Japanese Society for Neuroimmunology	Japan	Literature review	6/7
