# Supplementary material for: Development of a Model Care Pathway for Myasthenia Gravis
Source: Int J Environ Res Public Health. 2021 Nov 4;18(21):11591. doi: 10.3390/ijerph182111591 (PMC8582978; doi:10.3390/ijerph182111591)
Supplement: Supplementary file 1 [file ijerph-18-11591-s001.zip › S4_Characterstics of included reviews and other documents.pdf]

S4 - Characteristics of included reviews and other documents.

N	Title	First author	Publication year	Topics
1	The myasthenic patient in crisis: an update of the management in Neuro-intensive Care Unit	Daniel Agustin Godoy	2013	Process flow/algorithm presented for the Pathophysiology, the management of MG crisis
2	Treatment of Myasthenia Gravis	Constantine Farmakidis	2018	Algorithms presented for the treatment of generalized MG and myasthenic crisis.
3	Myasthenia gravis	Nils Erik Gilhus	2019	Algorithms presented for MG Diagnosis, chronic and acute treatment.
4	Myasthenia gravis: subgroup classification and therapeutic strategies	Nils Erik Gilhus	2015	Care pathway presented for treatment of Generalized MG and MG crisis
5	Myasthenia Gravis	Nils Erik Gilhus	2016	Algorithms presented for the treatment of chronic and acute Generalized MG
6	When myasthenia gravis is deemed refractory: clinical signposts and treatment strategies	Renato Mantegazza	2017	The algorithm presented for the treatment of MG
7	Diagnosis and management of myasthenia gravis	Sivakumar Sathasivam	2014	Process flows/algorithms presented for the diagnosis and treatment of Generalized MG and MG crisis
8	Myasthenia Gravis – A Review of Current Therapeutic Options	Saiju Jacob	2018	The algorithm presented for the treatment MG
9	Standard operating procedure (SOP) myasthenic crisis	Henning Stetefeld	2019	Process flows/algorithms presented for the standard operating procedure of MG crisis
10	Treatment of Myasthenia Gravis Based on Its Immunopathogenesis	Jee Young Kim	2011	Process flow/Care pathway presented for management of MG
11	Autoimmune myasthenia gravis: emerging clinical and biological heterogeneity	Matthew N Meriggioli	2009	Process flow/algorithm presented for diagnosis and treatment for MG
12	Outcome Measures in Myasthenia Gravis: Incorporation into Clinical Practice	Srikanth Muppidi	2017	Outcome measure instruments for Myasthenia gravis
13	Myasthenia gravis: new developments in research and treatment	Amelia Evoli	2017	Benchmarking information for MG from the EURO-NMD network.
14	Myasthenia Gravis: MGFA Manual for the Health Care Provider	James F Howard	2009	Multi-disciplinary practice recommendations for MG
