# Supplementary material for: Development of a Model Care Pathway for Myasthenia Gravis
Source: Int J Environ Res Public Health. 2021 Nov 4;18(21):11591. doi: 10.3390/ijerph182111591 (PMC8582978; doi:10.3390/ijerph182111591)
Supplement: Supplementary file 1 [file ijerph-18-11591-s001.zip › S6_Detailed description of a Key intervention.pdf]

S6- Example of a detailed description of a key intervention: Acetylcholinesterase inhibitor for symptomatic treatment.

Argumentation

Acetylcholinesterase inhibitor (Pyridostigmine) is essential for treating the symptoms for all sub-groups of MG patients except in patients with anti-MuSK antibodies. It should be prescribed as the initial treatment when the diagnosis is confirmed. The dose is adjusted according to the symptoms of the patients. These inhibitors are well tolerated, fast-acting, safe, and free of long-term side effects. In patients with ocular myasthenia, these inhibitors have a good effect on ptosis than on diplopia and do not influence the development of generalization. International guidelines recommended these inhibitors for the symptomatic treatment, based mainly on the expert consensus and a little number of controlled studies. *(Sanders et al., 2016; Kerty et al., 2014; Skeie et al., 2010; Sussman et al., 2015)*.

In the Delphi study, 99% of experts were agreed that the key intervention had a high impact on clinical outcomes and therefore should be included in the pathway. Moreover, experts ranked as first which means that this key intervention has higher importance in the treatment.

Level of evidence

Class A evidence

Description

Pyridostigmine is initially started with the dosage of 30 mg 3-4 times daily for 2-4 days and that can be increased to 60 mg 4-5 times daily for 5 days, based on the clinical effect and patient tolerance. For pediatric patients, the standard dose is 7 mg/kg/day. If the symptoms are controlled the patients can continue the treatment, if not switch to corticosteroids and/or other immunosuppressive therapy. *(Sanders et al., 2016; Kerty et al., 2014; Skeie et al., 2010; Sussman et al., 2015)*

References

Sanders, D. B., Wolfe, G. I., Benatar, M., Evoli, A., Gilhus N.E., Illa, I., Kuntz, N., Massey, J.M., Melms, A., Murai, H., Nicolle, M., Palace, J., BM, Richman, D.P., Verschuuren, J., Narayanaswami, P. (2016). International Consensus Guidance for the Management of Myasthenia Gravis. American Academy of Neurology.

Kerty, E., Elsais, A., Argov, Z., Evolid, A., Gilhuse, N. E. (2014). EFNS/ENS Guidelines for the treatment of ocular myasthenia. European Journal of Neurology, 21: 687–693.

Skeie, G. O., Apostolski, S., Evoli, A., Gilhus, N. E., Illa, I., Harms, L., Hilton-Jones, D., Melms, A., Verschuuren, J., Horge, H. W. (2010). Guidelines for treatment of autoimmune neuromuscular transmission disorders. European Journal of Neurology, 17: 893–902.

Sussman, J., Farrugia, M. E., Maddison, P., Hill, M., Leite, M. I., Jones, D. H. (2015). Myasthenia gravis: Association of British Neurologists' management guidelines. Pract Neurol, 15:199–206.
