# Supplementary material for: Development of a Model Care Pathway for Myasthenia Gravis
Source: Int J Environ Res Public Health. 2021 Nov 4;18(21):11591. doi: 10.3390/ijerph182111591 (PMC8582978; doi:10.3390/ijerph182111591)
Supplement: Supplementary file 1 [file ijerph-18-11591-s001.zip › S7_Detailed description of a Process indicator.pdf]

S7- Example of a detailed description of a process indicator.

**Performance measure Name:**	Appropriateness of initial therapy (Pyridostigmine)
**Description:**	These drugs prevent the breakdown of the neurotransmitter Ach (Acetylcholine) allowing more ACh to accumulate at the neuromuscular junction. ACh is the chemical compound that transmits nerve impulses to the muscle. Increased ACh improves the strength of muscles involved in eye movements, limb strength, swallowing, and breathing
**Rationale:**	International guidelines suggest that optimizing doses and timing of this therapy brings great advantages in the rapid resolution of symptoms
**Type of indicator:**	Process
**Improvement noted as:**	Increase in the rate of optimized prescribed therapies
**Numerator Statement:**	Number of adult patients with evidence of mild signs and symptoms and prescription of Pyridostigmine with a dose of 30–60mg every 4–8 hours<br>Included Population: Patients with a diagnosis of MG.<br>Excluded Population: None.
**Denominator Statement:**	Number of adult patients with evidence of mild signs and symptoms and prescription of Pyridostigmine<br>Included Population: Patients with a diagnosis of MG.<br>Exclude Population: None.
**Data collection method:**	Retrospective, patient records
**Data elements for the indicator:**	Evidence of Acetylcholinesterase-Inhibitors (AchE-I) therapy
**Data reported as:**	Percentage
**Expected performance:**	>95%
**Criteria to meet:**	All the therapy optimized
**References:**	Gilhus, N.E., Tzartos, S., Evoli, A. et al. Myasthenia gravis. Nat Rev Dis Primers 5, 30 (2019).<br><br>James F. Howard, Myasthenia Gravis Foundation of America Inc. (MGFA). A manual for Health Care Provider.<br><br>Bacci, E.D., Coyne, K.S., Poon, JL. et al. Understanding side effects of therapy for myasthenia gravis and their impact on daily life. BMC Neurol 19, 335 (2019). https://doi.org/10.1186/s12883-019-1573-2<br><br>Sanders DB, Wolfe GI, Benatar M, Evoli A, Gilhus NE, Illa I, Kuntz N, Massey JM, Melms A, Murai H, Nicolle M, Palace J, Richman DP, Verschuuren J, Narayanaswami P. International consensus guidance for management of myasthenia gravis: Executive summary. Neurology. 2016 Jul 26;87(4):419-25. doi: 10.1212/WNL.0000000000002790. Epub 2016 Jun 29. PMID: 27358333; PMCID: PMC4977114.<br><br>Narayanaswami P, Sanders DB, Wolfe G, Benatar M, Cea G, Evoli A, Gilhus NE, Illa I, Kuntz NL, Massey J, Melms A, Murai H, Nicolle M, Palace J, Richman D, Verschuuren J. International Consensus Guidance for Management of Myasthenia Gravis: 2020 Update. Neurology. 2021 Jan 19;96(3):114-122. doi: 10.1212/WNL.0000000000011124. Epub 2020 Nov 3. PMID: 33144515.
