# Supplementary figures and images for: Development of a Model Care Pathway for Myasthenia Gravis
Source: Int J Environ Res Public Health. 2021 Nov 4;18(21):11591. doi: 10.3390/ijerph182111591 (PMC8582978; doi:10.3390/ijerph182111591)

## Diagnosis

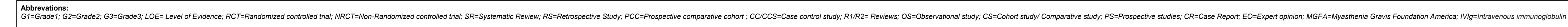

Supplement: Supplementary file 1 [file ijerph-18-11591-s001.zip › S5_Model pathway of MG.pdf]
